# Extended protocol and real-time PCR dataset for shrimp species identification

**DOI:** 10.1016/j.dib.2019.105068

**Published:** 2019-12-31

**Authors:** Debabrata Mondal, Nripendranath Mandal

**Affiliations:** Division of Molecular Medicine, Bose Institute, P-1/12 CIT Scheme VII-M, Kolkata, 700054, West Bengal, India

**Keywords:** Shrimp, Real-time PCR, Melt curve analysis, Species identification

## Abstract

Mitochondrial gene analysis was performed using gene-specific conserved primers by conventional and real-time PCR method. Along with, beta-actin gene analysis using conserved primers was also performed in this article. Mitochondrial gene sequence as well as beta-actin sequence data were acquired by Sanger sequencing technique. We have reported a set of conserved primers for the identification and authentication of ten different shrimp species. Real-time melt curve analysis was the most promising methodology to discriminate the mislabeling or fraudulent commercial use of shrimp species. Dataset in this article provided novel insight into identification and authentication of shrimp species by real-time melt curve analysis.

**Table:** Specifications Table

Subject	Aquaculture Biology
Specific subject area	Molecular Biology
Type of data	Table Image Figure
How data were acquired	Data were acquired by the conventional PCR and real-time PCR. Sequence data were acquired by Sanger sequencing technique.
Data format	Raw Analysed
Parameters for data collection	Real-time data and sequencing data were acquired by following the manufacturer's instruction and guidelines (Applied Biosystems, Massachusetts, USA).
Description of data collection	Real-time melt curve analysis was performed by five mitochondrial gene specific conserved primers. Beta-actin gene data were generated by conventional and real-time PCR using conserved primers. Gene insert containing purified plasmids were sequenced by Sanger sequencing technique, and analysed.
Data source location	Institution: Bose Institute City/Town/Region: Kolkata, West Bengal Country: India
Data accessibility	Data are with this article. Sanger sequencing data which were produced by Longer PCR method are available under the GenBank, NCBI: Accession ID: MK430836–MK430906. Small subunit ribosomal RNA (12SrRNA) gene sequences are available under the Accession IDs: MK430836.1 - MK430847.1 (https://www.ncbi.nlm.nih.gov/popset/?term=MK430836; https://www.ncbi.nlm.nih.gov/nuccore/MK430847). Large subunit ribosomal RNA (16SrRNA) gene sequences are available under the Accession IDs: MK430848.1 - MK430861.1 (https://www.ncbi.nlm.nih.gov/popset/?term=MK430848; https://www.ncbi.nlm.nih.gov/popset/?term=MK430860). Cytochrome oxidase subunit 1 (COX1) gene sequences are available under the Accession IDs: MK430862.1 - MK430879.1 (https://www.ncbi.nlm.nih.gov/popset/?term=MK430862; https://www.ncbi.nlm.nih.gov/nuccore/1720684370; https://www.ncbi.nlm.nih.gov/nuccore/MK430877; https://www.ncbi.nlm.nih.gov/nuccore/MK430878; https://www.ncbi.nlm.nih.gov/nuccore/MK430879). Cytochrome *b* (cytb) gene sequences are available under the Accession IDs: MK430880.1 - MK430897.1 (https://www.ncbi.nlm.nih.gov/popset/?term=MK430880; https://www.ncbi.nlm.nih.gov/nuccore/1720684402; https://www.ncbi.nlm.nih.gov/nuccore/MK430894; https://www.ncbi.nlm.nih.gov/nuccore/MK430895; https://www.ncbi.nlm.nih.gov/nuccore/MK430896; https://www.ncbi.nlm.nih.gov/nuccore/MK430897). NADH dehydrogenase subunit 1 (ND1) gene sequences are available under the Accession IDs: MK430898.1 - MK430906.1 (https://www.ncbi.nlm.nih.gov/popset/?term=MK430898; https://www.ncbi.nlm.nih.gov/nuccore/1720684425). ND1 gene sequences of *Marsupenaeus japonicus* and *Parapenaeopsis uncta* will be available under the Accession IDs: GenBank MN782515 - MN782517. Beta-actin gene sequences will be available under the Accession IDs: GenBank MN784945 - MN784955.
Related research article	Author's name: Debabrata Mondal, Nripendranath Mandal Title: Molecular phylogeny of mitochondrial DNA: Shrimp species identification by multiplex and real-time PCR Journal: Food Control Volume: 108 DOI: https://doi.org/10.1016/j.foodcont.2019.106868 [1]

**Table:** 

**Value of the Data** • The extended dataset would be extremely valuable to prevent the fraudulent commercial use of shrimp species. • Dataset in this article offer novel insight into identification and authentication of shrimp species by real-time PCR to researchers. • The data in this article can be used in future to identify new shrimp species. • Dataset in this article will provide clarity to gene-specific amplicons generated by multiplex PCR and real-time PCR.

## 1 Data

**Fig. 1:**
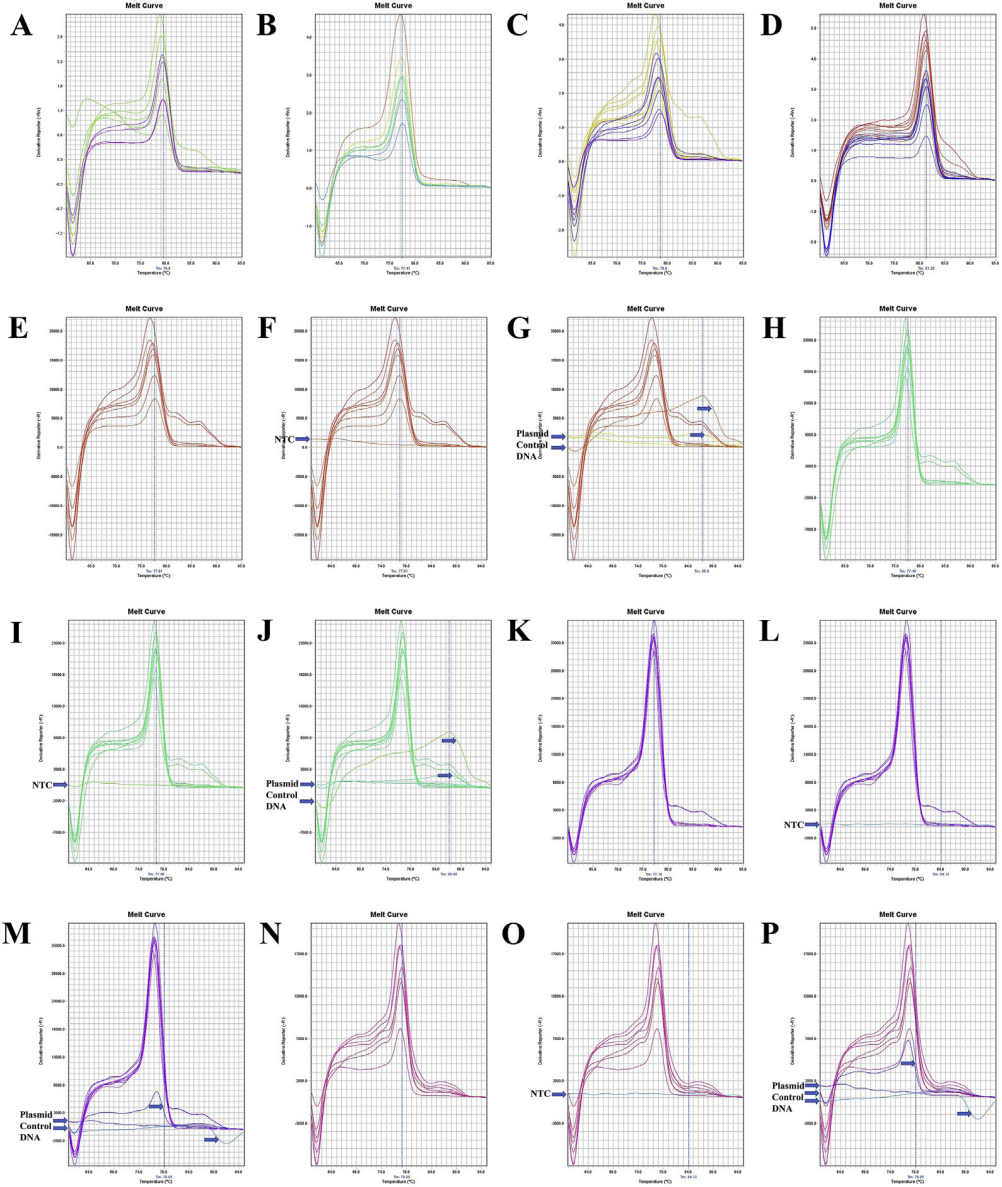
A. The melt curve of COX1 gene of *L. vannamei* amplified by RTCOX2F(M) – RTCOX2R(M) primer. B. The melt curve of COX1 gene of *M. rosenbergii* amplified by RTCOX2F(M) – RTCOX2R(M) primer. C. The melt curve of COX1 gene of *P. hardwickii* amplified by RTCOX2F(M) – RTCOX2R(M) primer. D. The melt curve of COX1 gene of *P. uncta* amplified by RTCOX2F(M) – RTCOX2R(M) primer. E. The melt curve of COX1 gene of *P. monodon* amplified by RTCOX2F(M) – RTCOX2R(M) primer. F. The melt curve of COX1 gene of *P. monodon* with NTC (No Template Control) amplified by RTCOX2F(M) – RTCOX2R(M) primer. G. The melt curve of COX1 gene of *P. monodon* with Plasmid Control DNA 1 [circular supercoiled plasmid vector pTZ57R DNA without insert, InsTAclone PCR Cloning Kit, Cat#K1213 or #K1214; concentration used in real-time PCR: 100 ng (stock: 50 ng/µL), 20 ng (stock: 10 ng/µL), and 4 ng (stock: 2 ng/µL)] amplified by RTCOX2F(M) – RTCOX2R(M) primer. H. The melt curve of COX1 gene of *P. monodon* amplified by RTCOX2F(M) – RTCOX2R(N) primer. I. The melt curve of COX1 gene of *P. monodon* with NTC amplified by RTCOX2F(M) – RTCOX2R(N) primer. J. The melt curve of COX1 gene of *P. monodon* with Plasmid Control DNA 1 amplified by RTCOX2F(M) – RTCOX2R(N) primer. K. The melt curve of COX1 gene of *P. monodon* amplified by RTCOX2F(N) – RTCOX2R(N) primer. L. The melt curve of COX1 gene of *P. monodon* with NTC amplified by RTCOX2F(N) – RTCOX2R(N) primer. M. The melt curve of COX1 gene of *P. monodon* with Plasmid Control DNA 1 amplified by RTCOX2F(N) – RTCOX2R(N) primer. N. The melt curve of COX1 gene of *S. crassicornis* amplified by RTCOX2F(N) – RTCOX2R(N) primer. O. The melt curve of COX1 gene of *S. crassicornis* with NTC amplified by RTCOX2F(N) – RTCOX2R(N) primer. P. The melt curve of COX1 gene of *S. crassicornis* with Plasmid Control DNA 1 amplified by RTCOX2F(N) – RTCOX2R(N) primer.

**Fig. 2:**
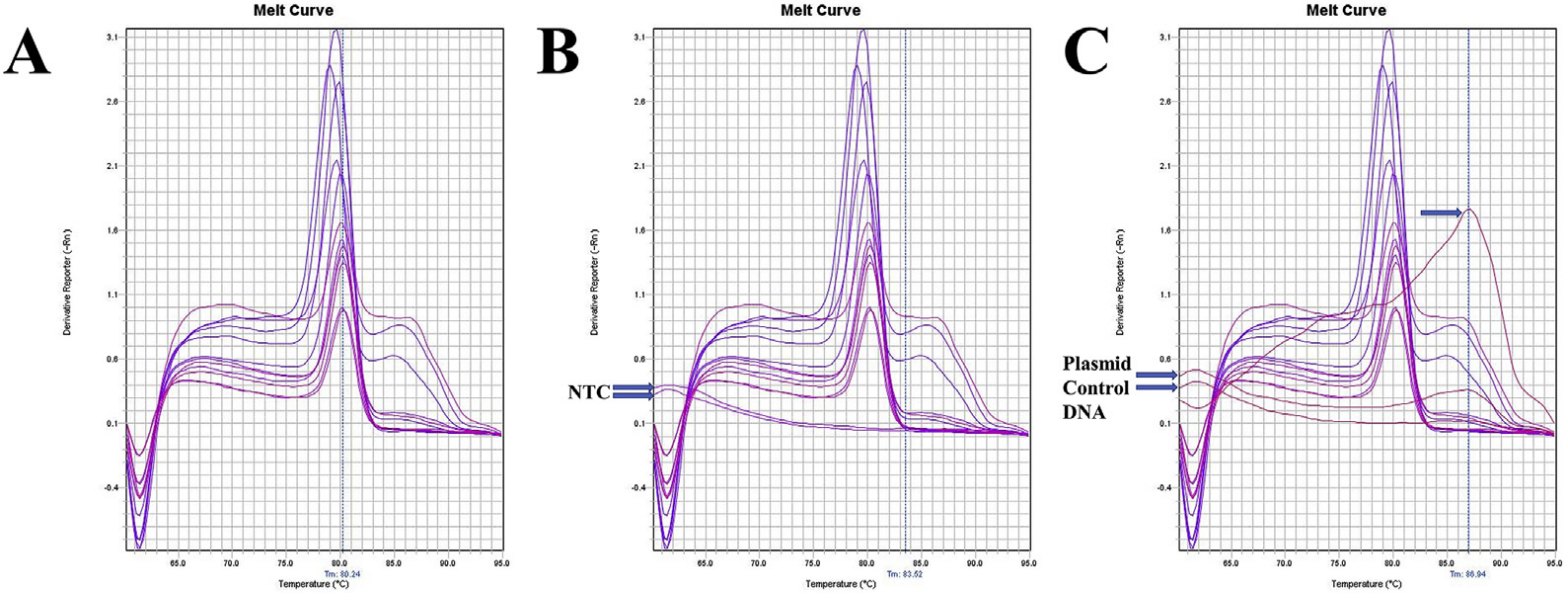
A. The melt curve of Cytb gene of *F. merguiensis* amplified by RTCytbF1 – RTCytbR1 primer. B. The melt curve of Cytb gene of *F. merguiensis* with NTC amplified by RTCytbF1 – RTCytbR1 primer. C. The melt curve of Cytb gene of *F. merguiensis* with Plasmid Control DNA 1 amplified by RTCytbF1 – RTCytbR1 primer.

**Fig. 3:**
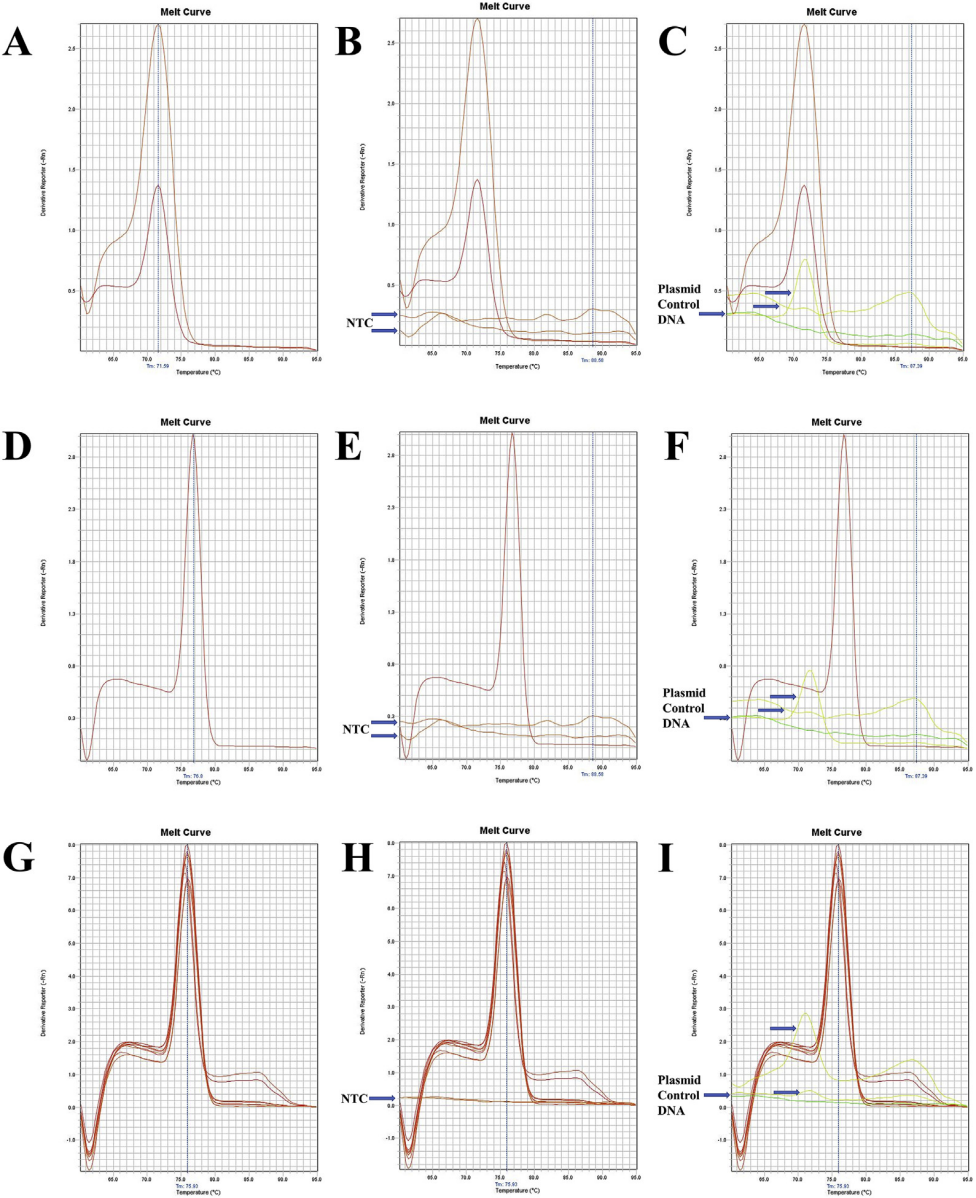
The melt curve patterns of 16SrRNA gene of *L. vannamei*. A. The melt curve of 16SrRNA gene of *L. vannamei* amplified by RT16Sf(N) - RT16Sr primer. B. The melt curve of 16SrRNA gene of *L. vannamei* with NTC amplified by RT16Sf(N) - RT16Sr primer. C. The melt curve of 16SrRNA gene of *L. vannamei* with Plasmid Control DNA 1 amplified by RT16Sf(N) - RT16Sr primer. D. The melt curve of 16SrRNA gene of *L. vannamei* amplified by RT16Sf(N) - RT16Sr primer. E. The melt curve of 16SrRNA gene of *L. vannamei* with NTC amplified by RT16Sf(N) - RT16Sr primer. F. The melt curve of 16SrRNA gene of *L. vannamei* with Plasmid Control DNA 1 amplified by RT16Sf(N) - RT16Sr primer. G. The melt curve of 16SrRNA gene of *L. vannamei* amplified by RT16Sf(N) - RT16Sr primer. H. The melt curve of 16SrRNA gene of *L. vannamei* with NTC amplified by RT16Sf(N) - RT16Sr primer. I. The melt curve of 16SrRNA gene of *L. vannamei* with Plasmid Control DNA 1 amplified by RT16Sf(N) - RT16Sr primer.

**Fig. 4:**
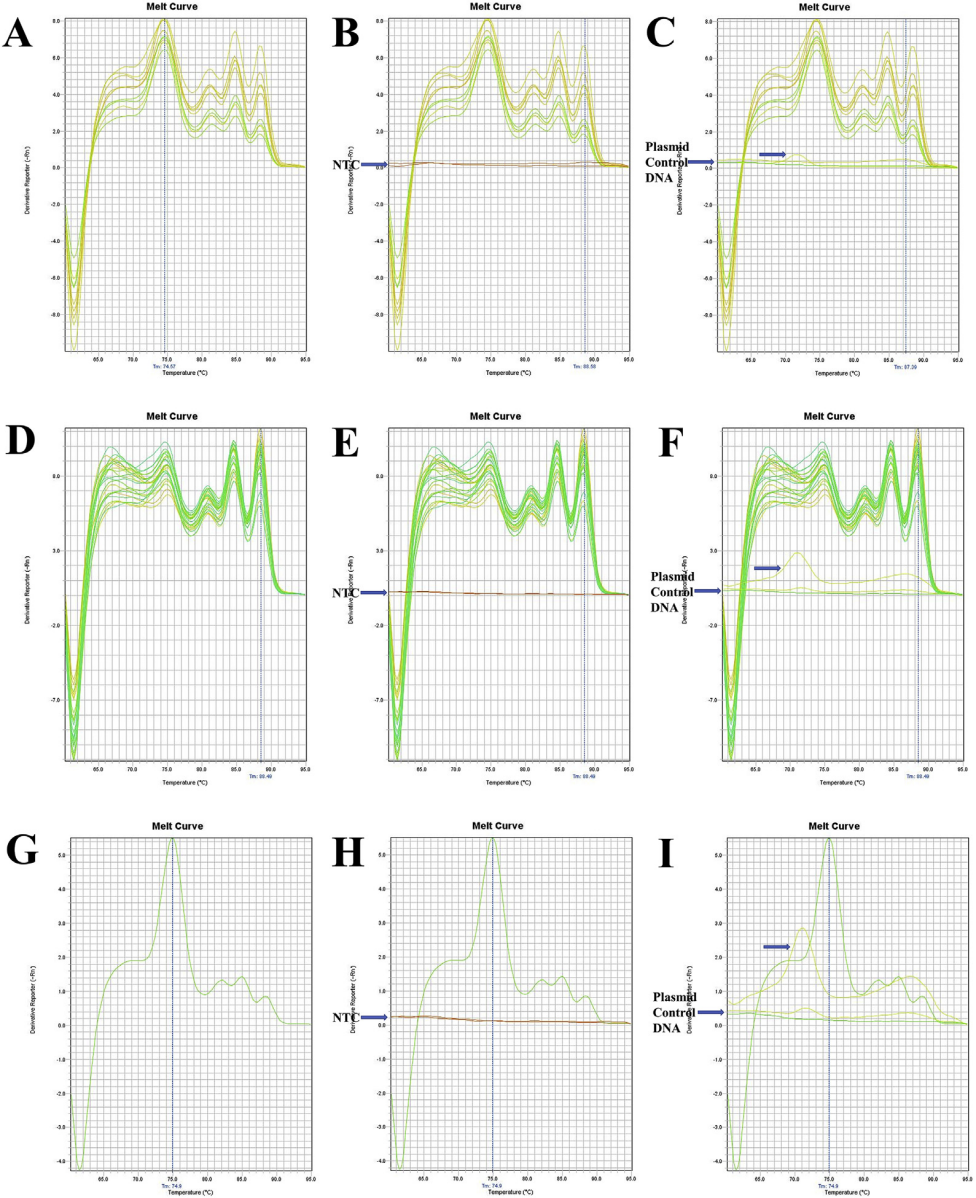
The melt curve patterns of 16SrRNA gene of *M. japonicus*. A. The melt curve of 16SrRNA gene of *M. japonicus* amplified by RT16Sf(N) - RT16Sr primer. B. The melt curve of 16SrRNA gene of *M. japonicus* with NTC amplified by RT16Sf(N) - RT16Sr primer. C. The melt curve of 16SrRNA gene of *M. japonicus* with Plasmid Control DNA 1 amplified by RT16Sf(N) - RT16Sr primer. D. The melt curve of 16SrRNA gene of *M. japonicus* amplified by RT16Sf(N) - RT16Sr primer. E. The melt curve of 16SrRNA gene of *M. japonicus* with NTC amplified by RT16Sf(N) - RT16Sr primer. F. The melt curve of 16SrRNA gene of *M. japonicus* with Plasmid Control DNA 1 amplified by RT16Sf(N) - RT16Sr primer. G. The melt curve of 16SrRNA gene of *M. japonicus* amplified by RT16Sf(N) - RT16Sr primer. H. The melt curve of 16SrRNA gene of *M. japonicus* with NTC amplified by RT16Sf(N) - RT16Sr primer. I. The melt curve of 16SrRNA gene of *M. japonicus* with Plasmid Control DNA 1 amplified by RT16Sf(N) - RT16Sr primer.

**Fig. 5:**
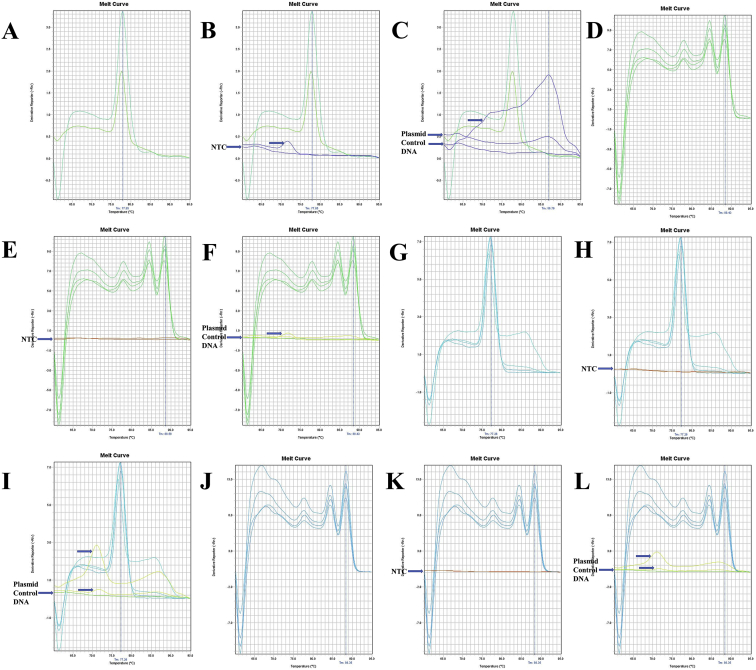
The melt curve patterns of 16SrRNA gene of *M. rosenbergii*. A. The melt curve of 16SrRNA gene of *M. rosenbergii* amplified by RT16Sf(N) - RT16Sr primer (annealing temp.: 55 °C). B. The melt curve of 16SrRNA gene of *M. rosenbergii* with NTC amplified by RT16Sf(N) - RT16Sr primer (annealing temp.: 55 °C). C. The melt curve of 16SrRNA gene of *M. rosenbergii* with Plasmid Control DNA 1 amplified by RT16Sf(N) - RT16Sr primer (annealing temp.: 55 °C). D. The melt curve of 16SrRNA gene of *M. rosenbergii* amplified by RT16Sf(N) - RT16Sr primer (annealing temp.: 60 °C). E. The melt curve of 16SrRNA gene of *M. rosenbergii* with NTC amplified by RT16Sf(N) - RT16Sr primer (annealing temp.: 60 °C). F. The melt curve of 16SrRNA gene of *M. rosenbergii* with Plasmid Control DNA 1 amplified by RT16Sf(N) - RT16Sr primer (annealing temp.: 60 °C). G. The melt curve of 16SrRNA gene of *M. rosenbergii* amplified by RT16Sf(N) - RT16Sr primer (annealing temp.: 60 °C). H. The melt curve of 16SrRNA gene of *M. rosenbergii* with NTC amplified by RT16Sf(N) - RT16Sr primer (annealing temp.: 60 °C). I. The melt curve of 16SrRNA gene of *M. rosenbergii* with Plasmid Control DNA 1 amplified by RT16Sf(N) - RT16Sr primer (annealing temp.: 60 °C). J. The melt curve of 16SrRNA gene of *M. rosenbergii* amplified by RT16Sf(N) - RT16Sr primer (annealing temp.: 60 °C). K. The melt curve of 16SrRNA gene of *M. rosenbergii* with NTC amplified by RT16Sf(N) - RT16Sr primer (annealing temp.: 60 °C). L. The melt curve of 16SrRNA gene of *M. rosenbergii* with Plasmid Control DNA 1 amplified by RT16Sf(N) - RT16Sr primer (annealing temp.: 60 °C).

**Fig. 6:**
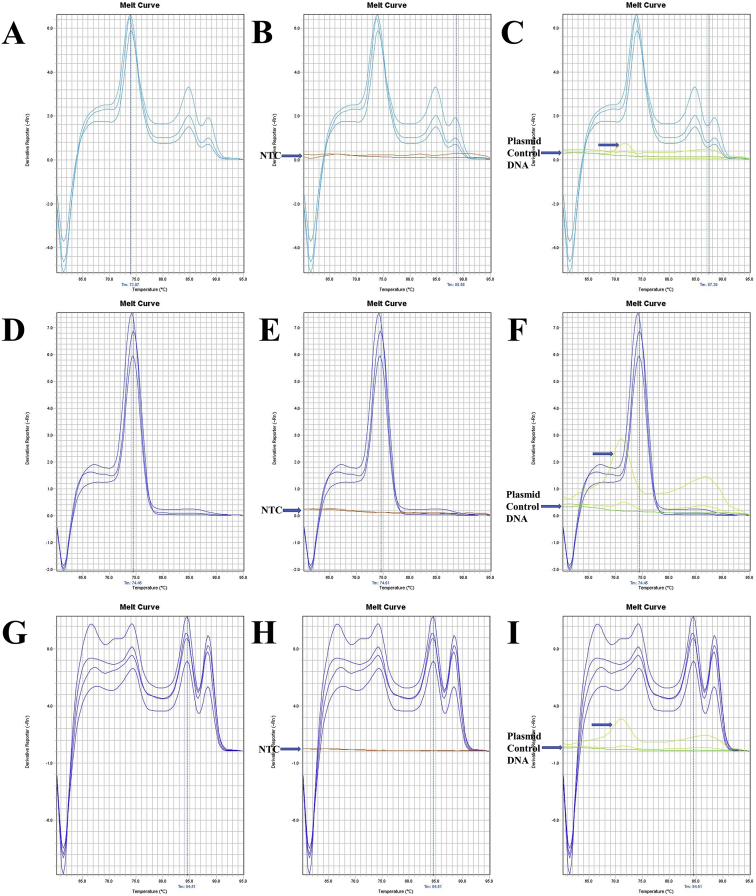
The melt curve patterns of 16SrRNA gene of *S. crassicornis*. A. The melt curve of 16SrRNA gene of *S. crassicornis* amplified by RT16Sf(N) - RT16Sr primer. B. The melt curve of 16SrRNA gene of *S. crassicornis* with NTC amplified by RT16Sf(N) - RT16Sr primer. C. The melt curve of 16SrRNA gene of *S. crassicornis* with Plasmid Control DNA 1 amplified by RT16Sf(N) - RT16Sr primer. D. The melt curve of 16SrRNA gene of *S. crassicornis* amplified by RT16Sf(N) - RT16Sr primer. E. The melt curve of 16SrRNA gene of *S. crassicornis* with NTC amplified by RT16Sf(N) - RT16Sr primer. F. The melt curve of 16SrRNA gene of *S. crassicornis* with Plasmid Control DNA 1 amplified by RT16Sf(N) - RT16Sr primer. G. The melt curve of 16SrRNA gene of *S. crassicornis* amplified by RT16Sf(N) - RT16Sr primer. H. The melt curve of 16SrRNA gene of *S. crassicornis* with NTC amplified by RT16Sf(N) - RT16Sr primer. I. The melt curve of 16SrRNA gene of *S. crassicornis* with Plasmid Control DNA 1 amplified by RT16Sf(N) - RT16Sr primer.

**Fig. 7:**
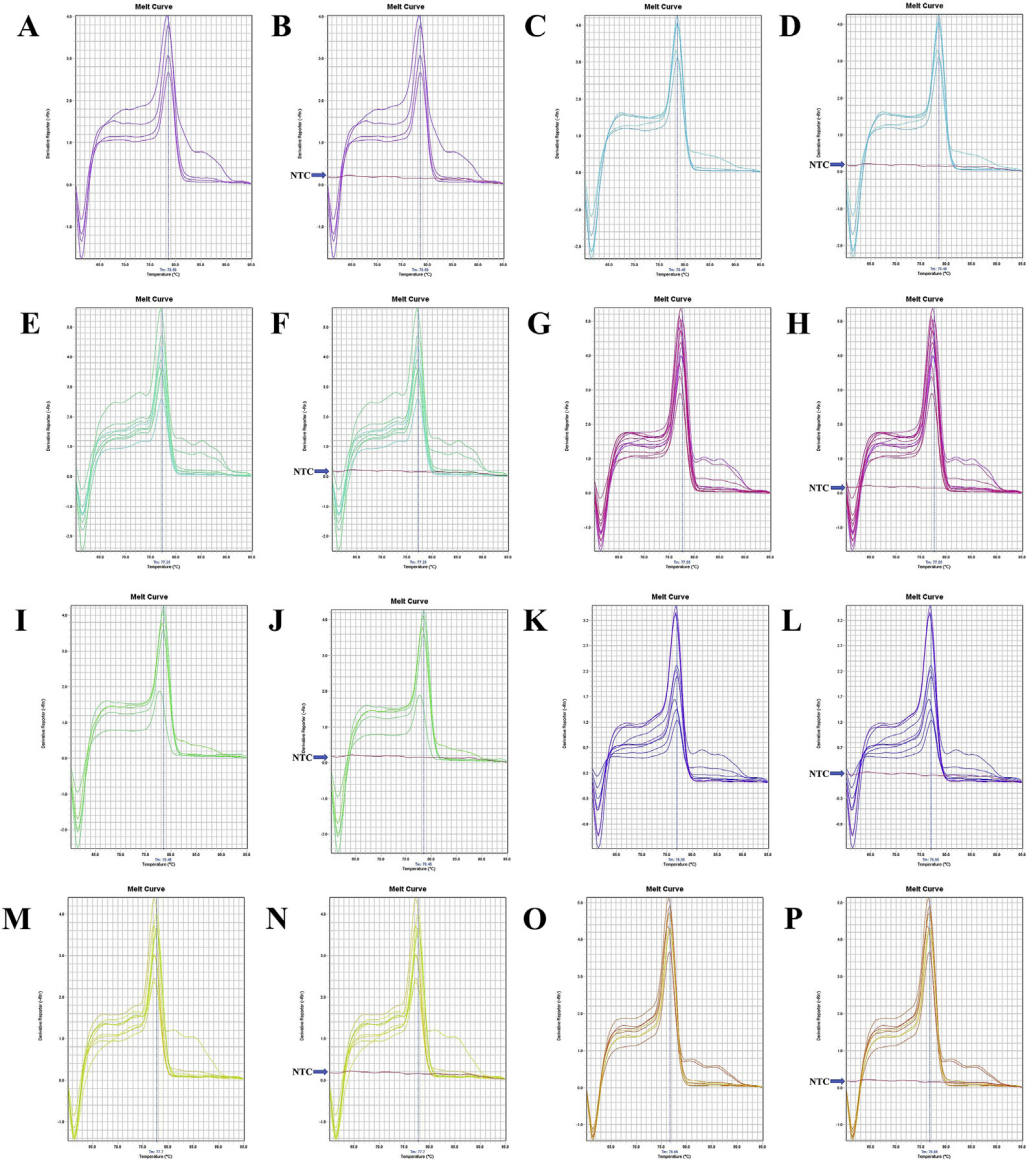
A. The melt curve of COX1 gene of *F. merguiensis* amplified by RTCOX2F(N) – RTCOX2R(N) primer. B. The melt curve of COX1 gene of *F. merguiensis* with NTC amplified by RTCOX2F(N) – RTCOX2R(N) primer. C. The melt curve of COX1 gene of *L. vannamei* amplified by RTCOX2F(N) – RTCOX2R(N) primer. D. The melt curve of COX1 gene of *L. vannamei* with NTC amplified by RTCOX2F(N) – RTCOX2R(N) primer. E. The melt curve of COX1 gene of *M. ensis* amplified by RTCOX2F(N) – RTCOX2R(N) primer. F. The melt curve of COX1 gene of *M. ensis* with NTC amplified by RTCOX2F(N) – RTCOX2R(N) primer. G. The melt curve of COX1 gene of *M. japonicus* amplified by RTCOX2F(N) – RTCOX2R(N) primer. H. The melt curve of COX1 gene of *M. japonicus* with NTC amplified by RTCOX2F(N) – RTCOX2R(N) primer. I. The melt curve of COX1 gene of *M. monoceros* amplified by RTCOX2F(N) – RTCOX2R(N) primer. J. The melt curve of COX1 gene of *M. monoceros* with NTC amplified by RTCOX2F(N) – RTCOX2R(N) primer. K. The melt curve of COX1 gene of *M. rosenbergii* amplified by RTCOX2F(N) – RTCOX2R(N) primer. L. The melt curve of COX1 gene of *M. rosenbergii* with NTC amplified by RTCOX2F(N) – RTCOX2R(N) primer. M. The melt curve of COX1 gene of *P. hardwickii* amplified by RTCOX2F(N) – RTCOX2R(N) primer. N. The melt curve of COX1 gene of *P. hardwickii* with NTC amplified by RTCOX2F(N) – RTCOX2R(N) primer. O. The melt curve of COX1 gene of *P. monodon* amplified by RTCOX2F(N) – RTCOX2R(N) primer. P. The melt curve of COX1 gene of *P. monodon* with NTC amplified by RTCOX2F(N) – RTCOX2R(N) primer.

**Fig. 8:**
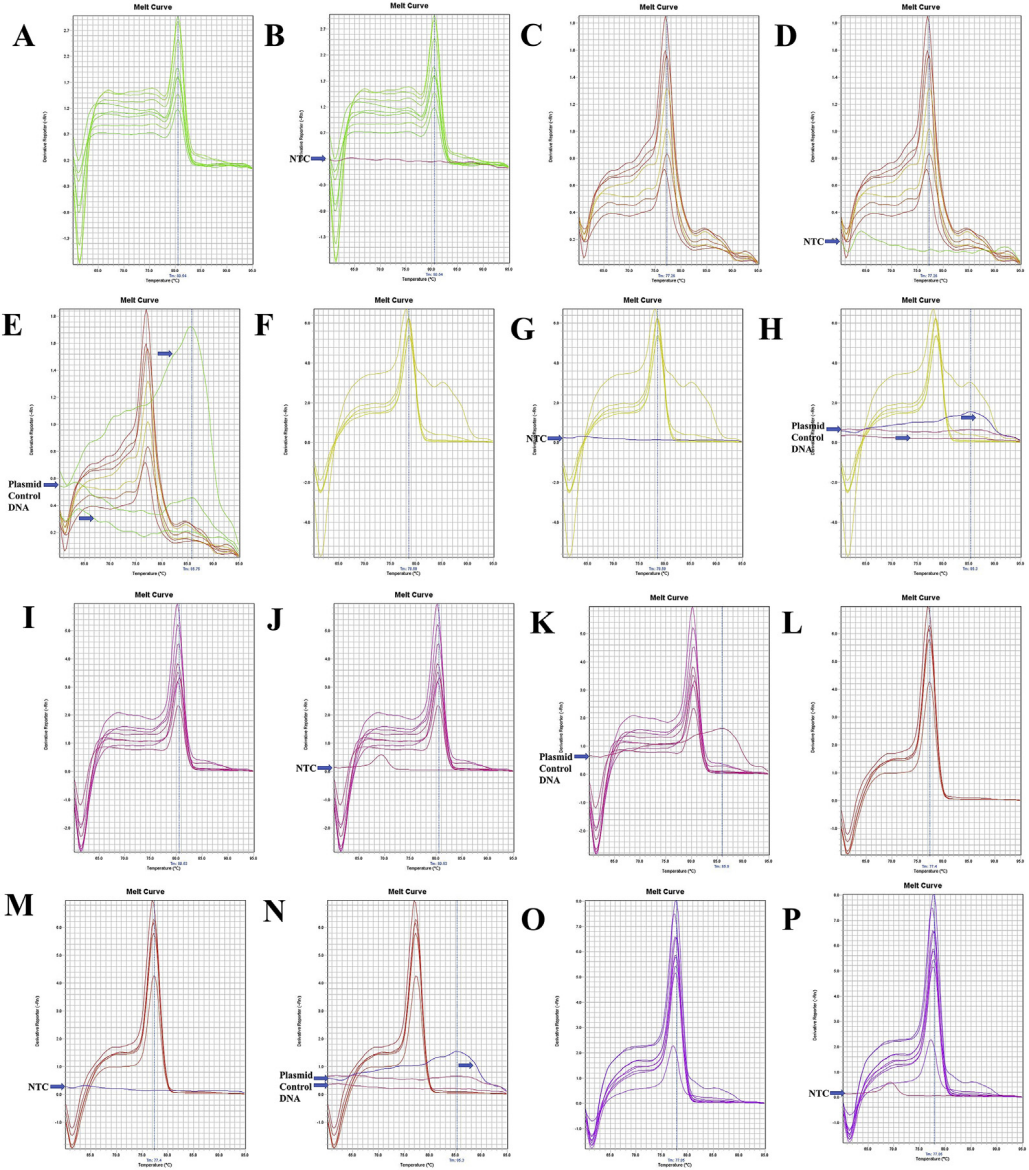
A. The melt curve of COX1 gene of *P. uncta* amplified by RTCOX2F(N) – RTCOX2R(N) primer. B. The melt curve of COX1 gene of *P. uncta* with NTC amplified by RTCOX2F(N) – RTCOX2R(N) primer. C. The melt curve of COX1 gene of *S. crassicornis* amplified by RTCOX2F(N) – RTCOX2R(N) primer. D. The melt curve of COX1 gene of *S. crassicornis* with NTC amplified by RTCOX2F(N) – RTCOX2R(N) primer. E. The melt curve of COX1 gene of *S. crassicornis* with Plasmid Control DNA 1 amplified by RTCOX2F(N) – RTCOX2R(N) primer. F. The melt curve of COX1 gene of *F. merguiensis* amplified by RTCOX1F(N) – RTCOX1R primer. G. The melt curve of COX1 gene of *F. merguiensis* with NTC amplified by RTCOX1F(N) – RTCOX1R primer. H. The melt curve of COX1 gene of *F. merguiensis* with Plasmid Control DNA 1 amplified by RTCOX1F(N) – RTCOX1R primer. I. The melt curve of COX1 gene of *L. vannamei* amplified by RTCOX1F(N) – RTCOX1R primer. J. The melt curve of COX1 gene of *L. vannamei* with NTC amplified by RTCOX1F(N) – RTCOX1R primer. K. The melt curve of COX1 gene of *L. vannamei* with Plasmid Control DNA 1 amplified by RTCOX1F(N) – RTCOX1R primer. L. The melt curve of COX1 gene of *L. vannamei* amplified by RTCOX1F(N) – RTCOX1R primer. M. The melt curve of COX1 gene of *L. vannamei* with NTC amplified by RTCOX1F(N) – RTCOX1R primer. N. The melt curve of COX1 gene of *L. vannamei* with Plasmid Control DNA 1 amplified by RTCOX1F(N) – RTCOX1R primer. O. The melt curve of COX1 gene of *M. ensis* amplified by RTCOX1F(N) – RTCOX1R primer. P. The melt curve of COX1 gene of *M. ensis* with NTC amplified by RTCOX1F(N) – RTCOX1R primer.

**Fig. 9:**
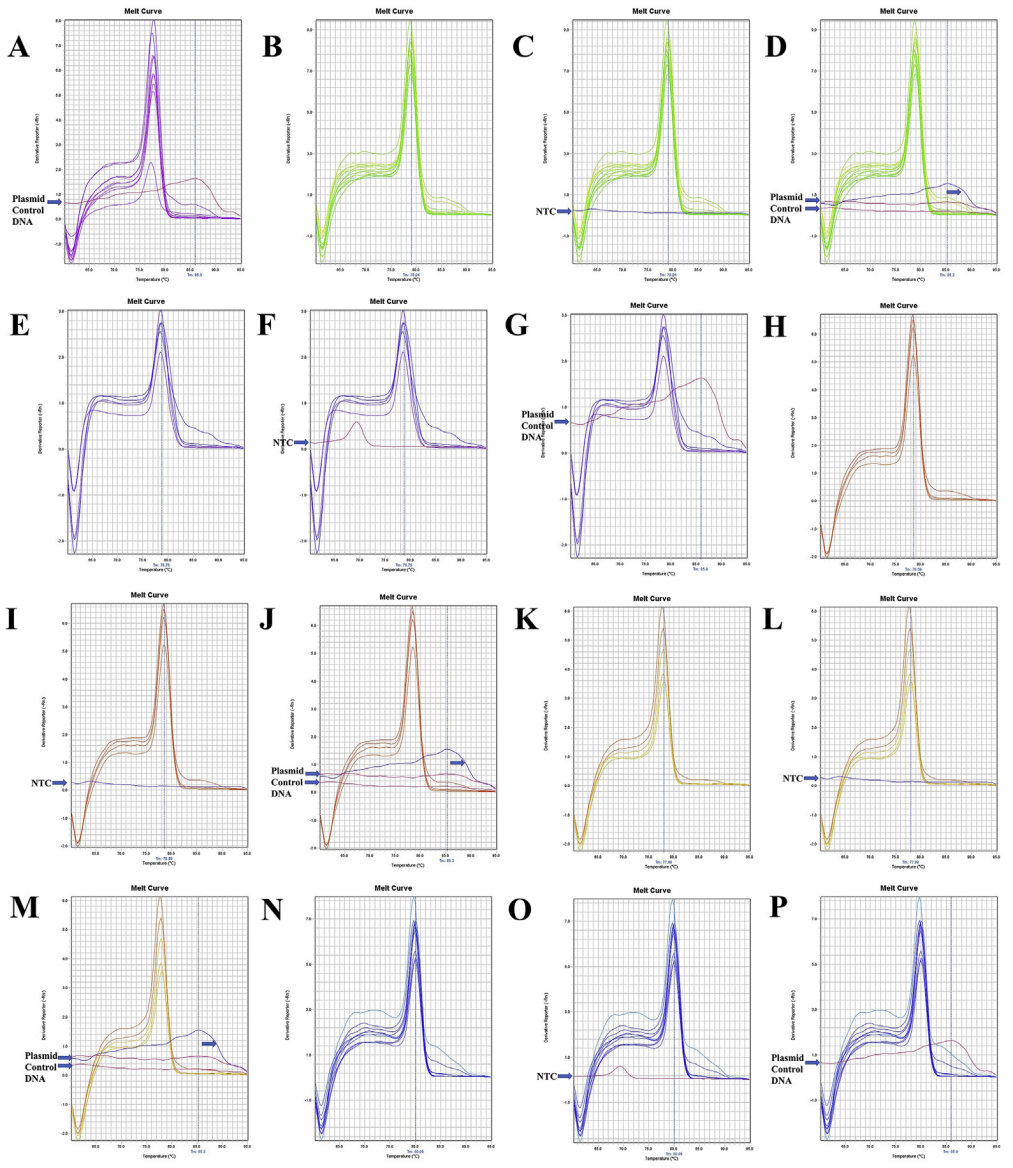
A. The melt curve of COX1 gene of *M. ensis* with Plasmid Control DNA 1 amplified by RTCOX1F(N) – RTCOX1R primer. B. The melt curve of COX1 gene of *M. japonicus* amplified by RTCOX1F(N) – RTCOX1R primer. C. The melt curve of COX1 gene of *M. japonicus* with NTC amplified by RTCOX1F(N) – RTCOX1R primer. D. The melt curve of COX1 gene of *M. japonicus* with Plasmid Control DNA 1 amplified by RTCOX1F(N) – RTCOX1R primer. E. The melt curve of COX1 gene of *M. monoceros* amplified by RTCOX1F(N) – RTCOX1R primer. F. The melt curve of COX1 gene of *M. monoceros* with NTC amplified by RTCOX1F(N) – RTCOX1R primer. G. The melt curve of COX1 gene of *M. monoceros* with Plasmid Control DNA 1 amplified by RTCOX1F(N) – RTCOX1R primer. H. The melt curve of COX1 gene of *M. rosenbergii* amplified by RTCOX1F(N) – RTCOX1R primer. I. The melt curve of COX1 gene of *M. rosenbergii* with NTC amplified by RTCOX1F(N) – RTCOX1R primer. J. The melt curve of COX1 gene of *M. rosenbergii* with Plasmid Control DNA 1 amplified by RTCOX1F(N) – RTCOX1R primer. K. The melt curve of COX1 gene of *M. rosenbergii* amplified by RTCOX1F(N) – RTCOX1R primer. L. The melt curve of COX1 gene of *M. rosenbergii* with NTC amplified by RTCOX1F(N) – RTCOX1R primer. M. The melt curve of COX1 gene of *M. rosenbergii* with Plasmid Control DNA 1 amplified by RTCOX1F(N) – RTCOX1R primer. N. The melt curve of COX1 gene of *P. uncta* amplified by RTCOX1F(N) – RTCOX1R primer. O. The melt curve of COX1 gene of *P. uncta* with NTC amplified by RTCOX1F(N) – RTCOX1R primer. P. The melt curve of COX1 gene of *P. uncta* with Plasmid Control DNA 1 amplified by RTCOX1F(N) – RTCOX1R primer.

**Fig. 10:**
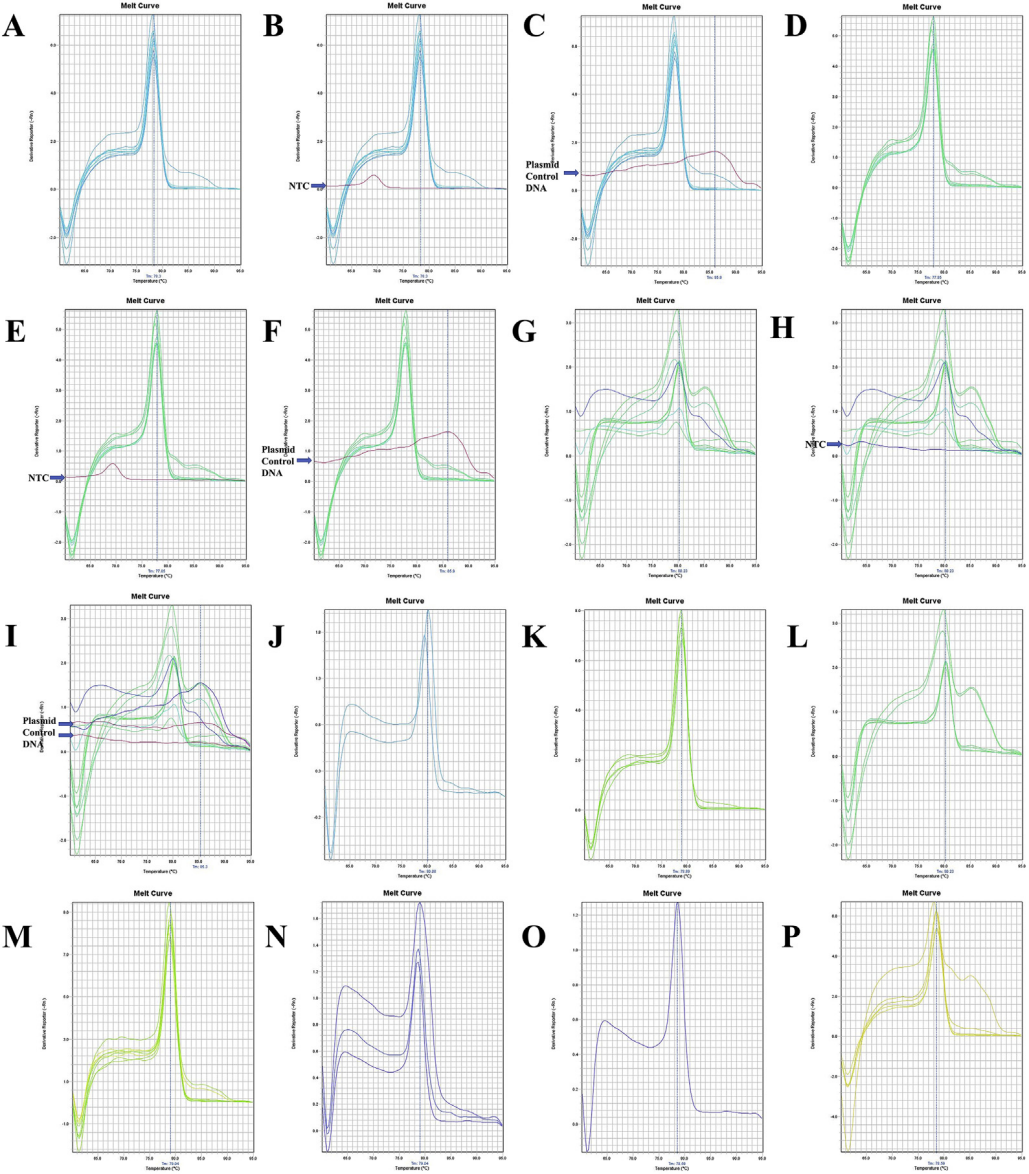
A. The melt curve of COX1 gene of *P. hardwickii* amplified by RTCOX1F(N) – RTCOX1R primer. B. The melt curve of COX1 gene of *P. hardwickii* with NTC amplified by RTCOX1F(N) – RTCOX1R primer. C. The melt curve of COX1 gene of *P. hardwickii* with Plasmid Control DNA 1 amplified by RTCOX1F(N) – RTCOX1R primer. D. The melt curve of COX1 gene of *P. monodon* amplified by RTCOX1F(N) – RTCOX1R primer. E. The melt curve of COX1 gene of *P. monodon* with NTC amplified by RTCOX1F(N) – RTCOX1R primer. F. The melt curve of COX1 gene of *P. monodon* with Plasmid Control DNA 1 amplified by RTCOX1F(N) – RTCOX1R primer. G. The melt curve of COX1 gene of *S. crassicornis* amplified by RTCOX1F(N) – RTCOX1R primer. H. The melt curve of COX1 gene of *S. crassicornis* with NTC amplified by RTCOX1F(N) – RTCOX1R primer. I. The melt curve of COX1 gene of *S. crassicornis* with Plasmid Control DNA 1 amplified by RTCOX1F(N) – RTCOX1R primer. J. The melt curve of COX1 gene of *F. merguiensis* amplified by RTCOX1F(M) – RTCOX1R primer. K. The melt curve of COX1 gene of *L. vannamei* amplified by RTCOX1F(M) – RTCOX1R primer. L. The melt curve of COX1 gene of *L. vannamei* amplified by RTCOX1F(M) – RTCOX1R primer. M. The melt curve of COX1 gene of *M. ensis* amplified by RTCOX1F(M) – RTCOX1R primer. N. The melt curve of COX1 gene of *M. japonicus* amplified by RTCOX1F(M) – RTCOX1R primer. O. The melt curve of COX1 gene of *M. japonicus* amplified by RTCOX1F(M) – RTCOX1R primer. P. The melt curve of COX1 gene of *M. monoceros* amplified by RTCOX1F(M) – RTCOX1R primer.

**Fig. 11:**
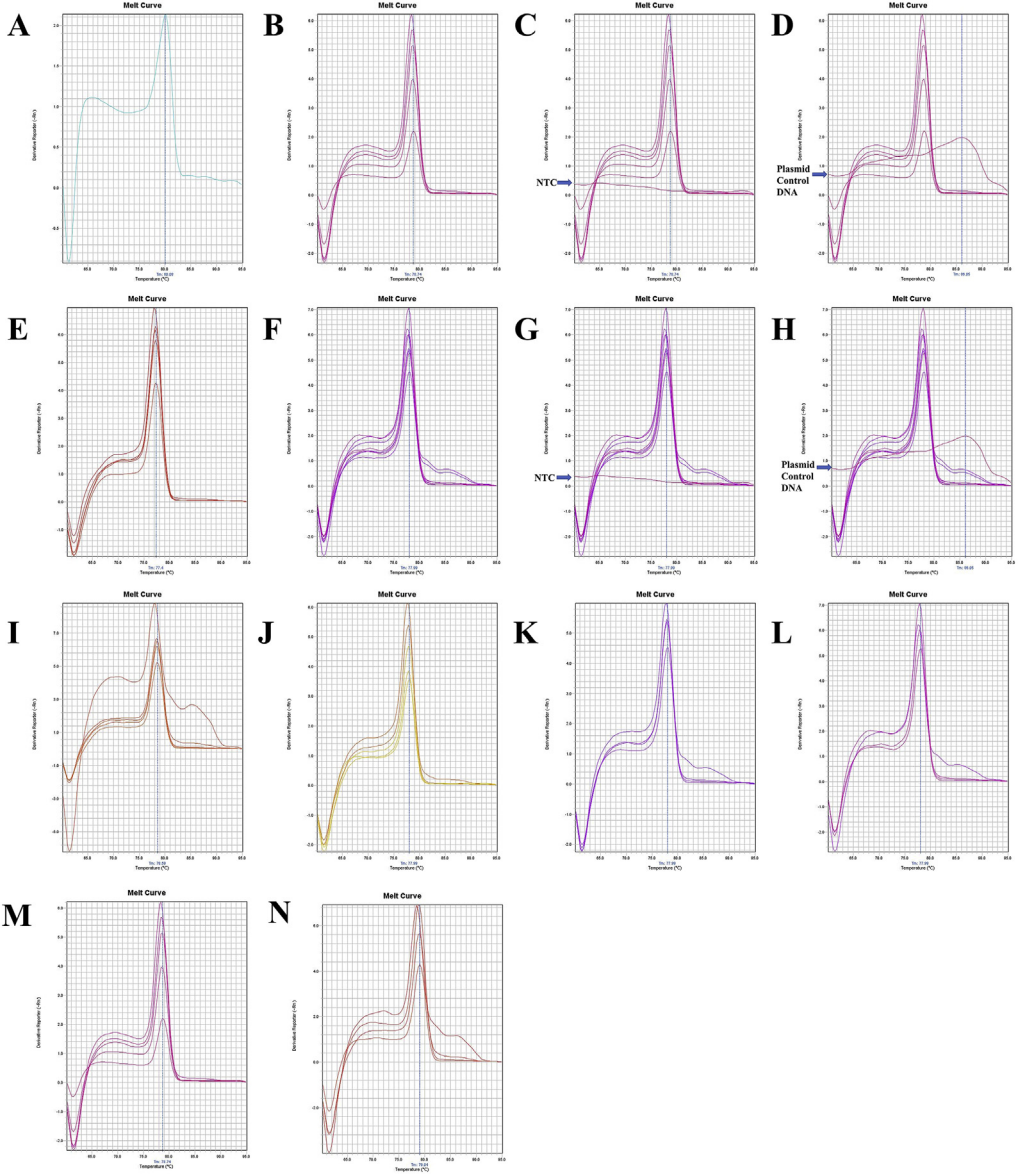
A. The melt curve of COX1 gene of *M. rosenbergii* amplified by RTCOX1F(M) – RTCOX1R primer. B. The melt curve of COX1 gene of *P. hardwickii* amplified by RTCOX1F(M) – RTCOX1R primer. C. The melt curve of COX1 gene of *P. hardwickii* with NTC amplified by RTCOX1F(M) – RTCOX1R primer. D. The melt curve of COX1 gene of *P. hardwickii* with Plasmid Control DNA 1 amplified by RTCOX1F(M) – RTCOX1R primer. E. The melt curve of COX1 gene of *P. hardwickii* amplified by RTCOX1F(M) – RTCOX1R primer. F. The melt curve of COX1 gene of *P. monodon* amplified by RTCOX1F(M) – RTCOX1R primer. G. The melt curve of COX1 gene of *P. monodon* with NTC amplified by RTCOX1F(M) – RTCOX1R primer. H. The melt curve of COX1 gene of *P. monodon* with Plasmid Control DNA 1 amplified by RTCOX1F(M) – RTCOX1R primer. I. The melt curve of COX1 gene of *P. uncta* amplified by RTCOX1F(M) – RTCOX1R primer. J. The melt curve of COX1 gene of *P. uncta* amplified by RTCOX1F(M) – RTCOX1R primer. K. The melt curve of COX1 gene of *S. crassicornis* amplified by RTCOX1F(M) – RTCOX1R primer. L. The melt curve of COX1 gene of *S. crassicornis* amplified by RTCOX1F(M) – RTCOX1R primer. M. The melt curve of COX1 gene of *S. crassicornis* amplified by RTCOX1F(M) – RTCOX1R primer. N. The melt curve of COX1 gene of *P. hardwickii* amplified by RTCOX1F(M) – RTCOX1R primer.

**Fig. 12:**
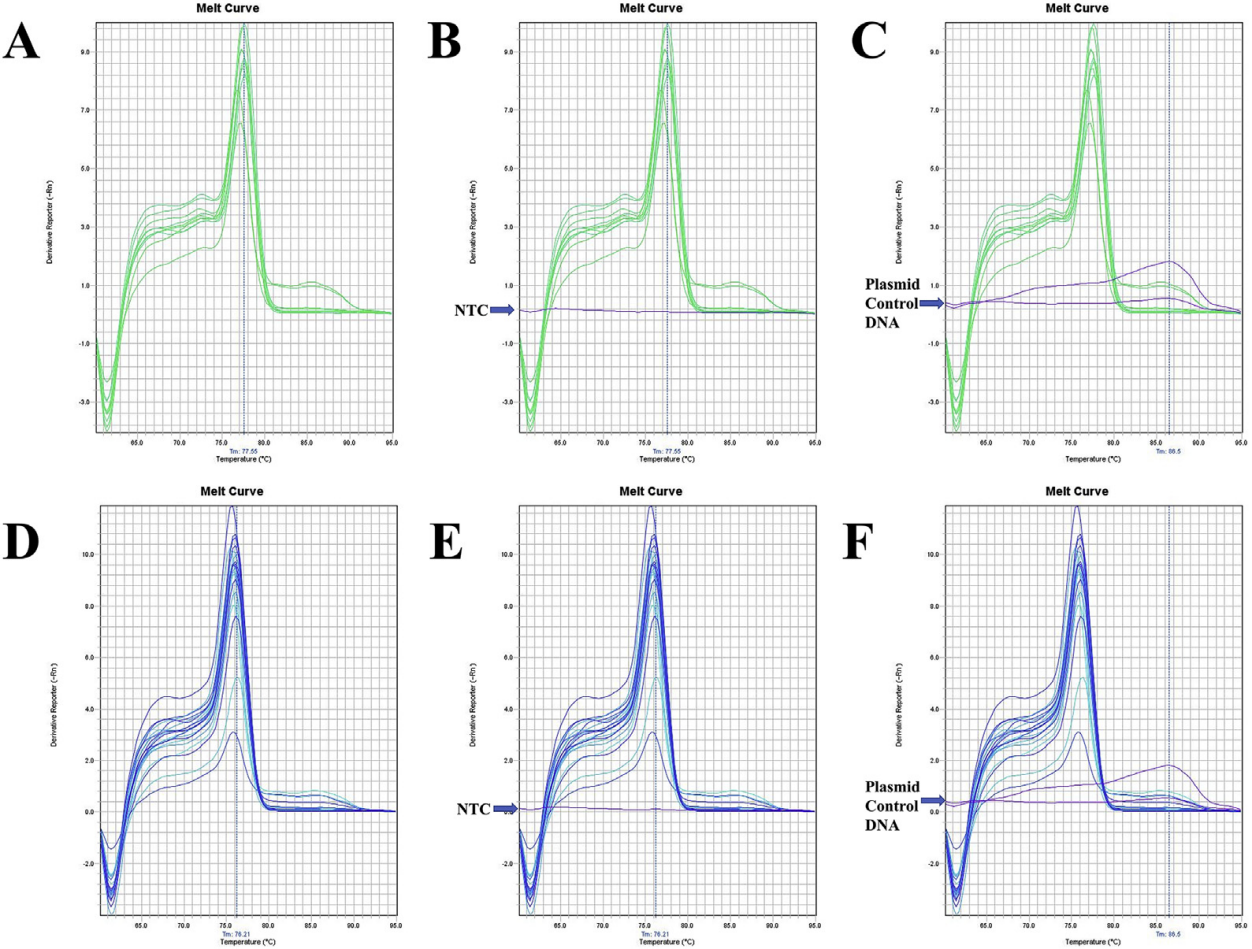
A. The melt curve of ND1 gene of *M. japonicus* amplified by RTND1F(N) – RTND1R primer. B. The melt curve of ND1 gene of *M. japonicus* with NTC amplified by RTND1F(N) – RTND1R primer. C. The melt curve of ND1 gene of *M. japonicus* with Plasmid Control DNA 1 amplified by RTND1F(N) – RTND1R primer. D. The melt curve of ND1 gene of *P. uncta* amplified by RTND1F(N) – RTND1R primer. E. The melt curve of ND1 gene of *P. uncta* with NTC amplified by RTND1F(N) – RTND1R primer. F. The melt curve of ND1 gene of *P. uncta* with Plasmid Control DNA 1 amplified by RTND1F(N) – RTND1R primer.

**Fig. 13:**
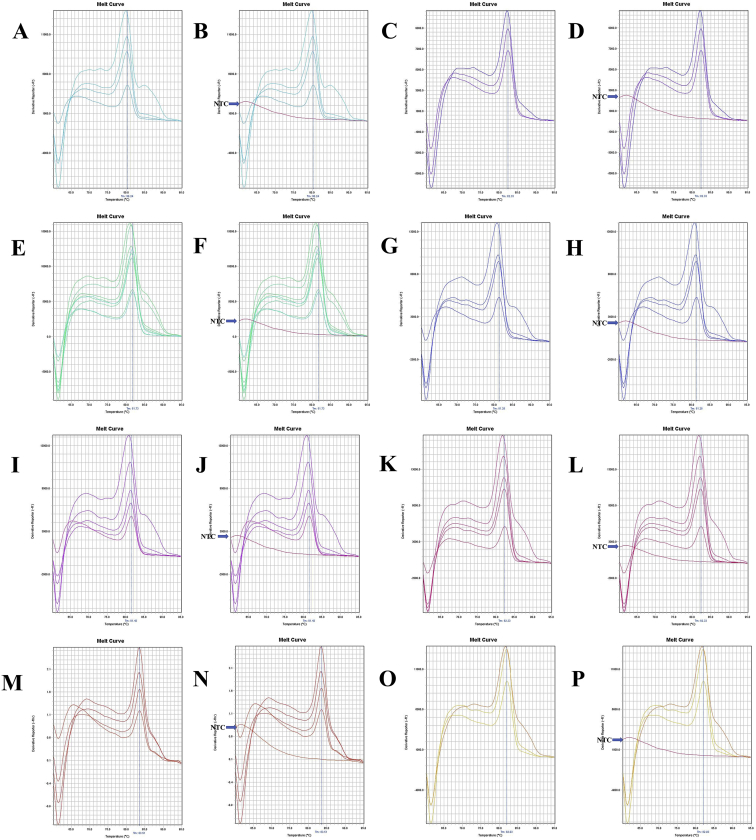
A. The melt curve of beta-actin gene of *F. merguiensis* amplified by B-act FE – B-act RS primer. B. The melt curve of beta-actin gene of *F. merguiensis* with NTC amplified by B-act FE – B-act RS primer. C. The melt curve of beta-actin gene of *L. vannamei* amplified by B-act FE – B-act RS primer. D. The melt curve of beta-actin gene of *L. vannamei* with NTC amplified by B-act FE – B-act RS primer. E. The melt curve of beta-actin gene of *M. ensis* amplified by B-act FE – B-act RS primer. F. The melt curve of beta-actin gene of *M. ensis* with NTC amplified by B-act FE – B-act RS primer. G. The melt curve of beta-actin gene of *M. japonicus* amplified by B-act FE – B-act RS primer. H. The melt curve of beta-actin gene of *M. japonicus* with NTC amplified by B-act FE – B-act RS primer. I. The melt curve of beta-actin gene of *M. monoceros* amplified by B-act FE – B-act RS primer. J. The melt curve of beta-actin gene of *M. monoceros* with NTC amplified by B-act FE – B-act RS primer. K. The melt curve of beta-actin gene of *M. rosenbergii* amplified by B-act FE – B-act RS primer. L. The melt curve of beta-actin gene of *M. rosenbergii* with NTC amplified by B-act FE – B-act RS primer. M. The melt curve of beta-actin gene of *M. rosenbergii* amplified by B-act FE – B-act RS primer. N. The melt curve of beta-actin gene of *M. rosenbergii* with NTC amplified by B-act FE – B-act RS primer. O. The melt curve of beta-actin gene of *P. hardwickii* amplified by B-act FE – B-act RS primer. P. The melt curve of beta-actin gene of *P. hardwickii* with NTC amplified by B-act FE – B-act RS primer.

**Fig. 14:**
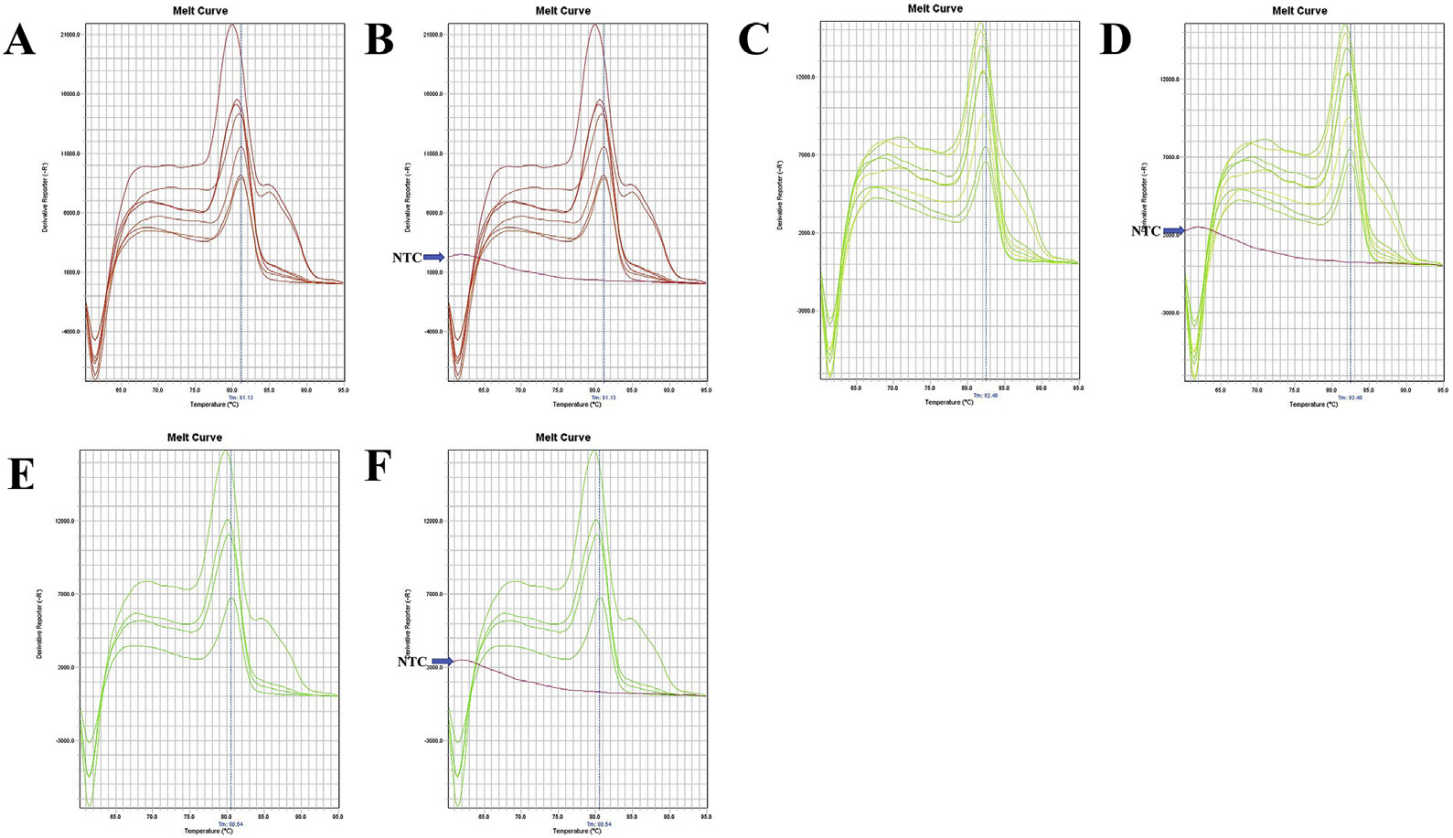
A. The melt curve of beta-actin gene of *P. monodon* amplified by B-act FE – B-act RS primer. B. The melt curve of beta-actin gene of *P. monodon* with NTC amplified by B-act FE – B-act RS primer. C. The melt curve of beta-actin gene of *P. uncta* amplified by B-act FE – B-act RS primer. D. The melt curve of beta-actin gene of *P. uncta* with NTC amplified by B-act FE – B-act RS primer. E. The melt curve of beta-actin gene of *S. crassicornis* amplified by B-act FE – B-act RS primer. F. The melt curve of beta-actin gene of *S. crassicornis* with NTC amplified by B-act FE – B-act RS primer.

**Fig. 15:**
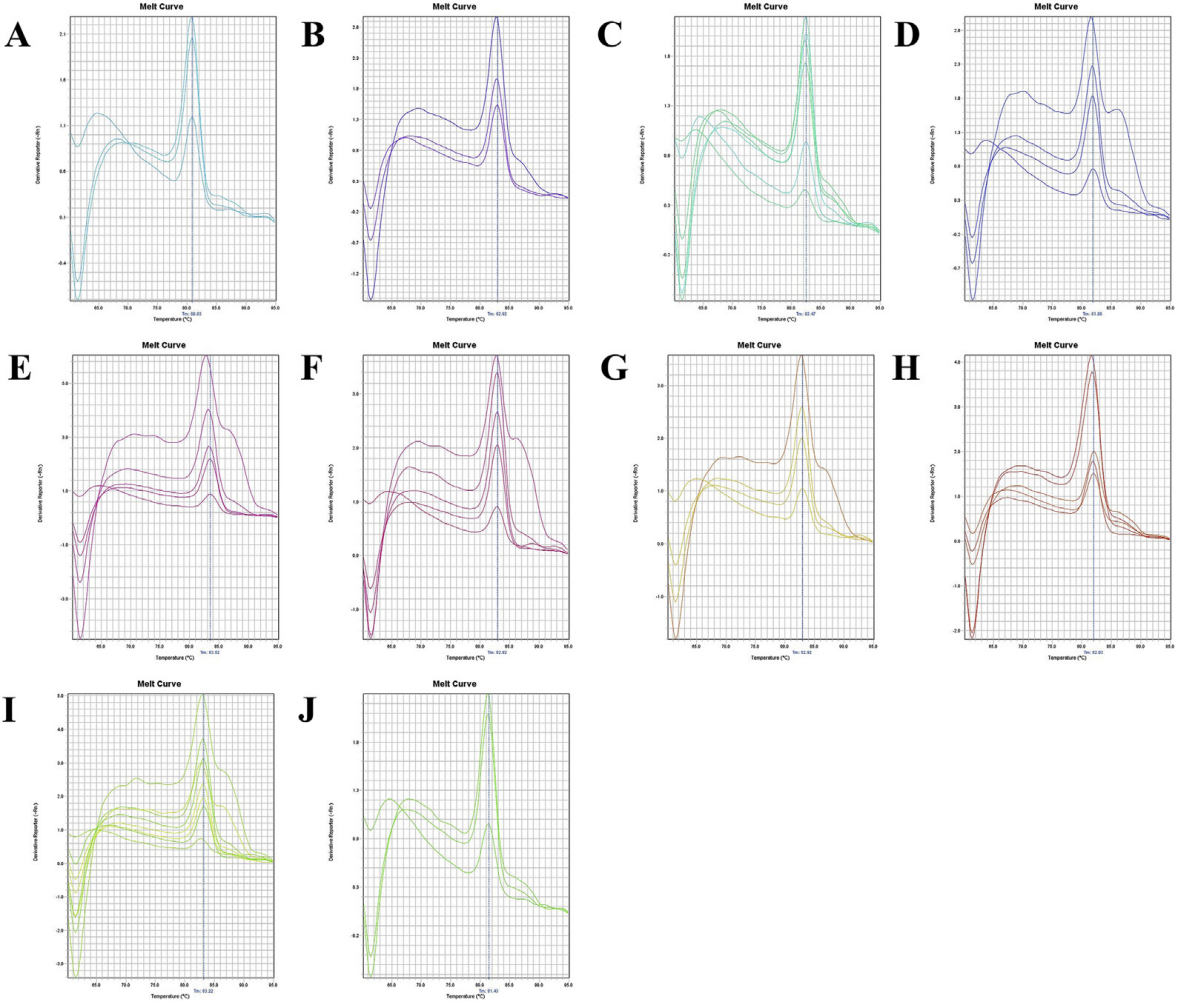
A. The melt curve of beta-actin gene of *F. merguiensis* amplified by B-act FE – B-act RS primer (annealing temp.: 60 °C). B. The melt curve of beta-actin gene of *L. vannamei* amplified by B-act FE – B-act RS primer (annealing temp.: 60 °C). C. The melt curve of beta-actin gene of *M. ensis* amplified by B-act FE – B-act RS primer (annealing temp.: 60 °C). D. The melt curve of beta-actin gene of *M. japonicus* amplified by B-act FE – B-act RS primer (annealing temp.: 60 °C). E. The melt curve of beta-actin gene of *M. monoceros* amplified by B-act FE – B-act RS primer (annealing temp.: 60 °C). F. The melt curve of beta-actin gene of *M. rosenbergii* amplified by B-act FE – B-act RS primer (annealing temp.: 60 °C). G. The melt curve of beta-actin gene of *P. hardwickii* amplified by B-act FE – B-act RS primer (annealing temp.: 60 °C). H. The melt curve of beta-actin gene of *P. monodon* amplified by B-act FE – B-act RS primer (annealing temp.: 60 °C). I. The melt curve of beta-actin gene of *P. uncta* amplified by B-act FE – B-act RS primer (annealing temp.: 60 °C). J. The melt curve of beta-actin gene of *S. crassicornis* amplified by B-act FE – B-act RS primer (annealing temp.: 60 °C).

The annealing temperature of five (12SrRNA, 16SrRNA, Cytochrome *c* oxidase subunit 1 or COX1, Cytochrome *b* or Cytb, and NADH Dehydrogenase Subunit 1 or ND1) mitochondrial gene specific conserved primers for longer-extension PCR were shown in Table 1. The gene fragments of ten shrimp species amplified by longer PCR were shown in Fig. S1. The beta-actin conserved primers were shown in Table 2, and PCR amplified fragments from ten shrimp species were shown in Fig. S2 and Table S3. The quality of the different gene insert containing purified plasmids was shown in Fig. S3. The ND1 gene sequence of *Marsupenaeus japonicus* or *Penaeus japonicus* and *Parapenaeopsis uncta* or *Ganjampenaeopsis uncta* was shown in Table S1. Mitochondrial gene sequence of different species was obtained by modifying the multiplex PCR method, and GeneDoc v. 2.7 [2] pairwise sequence alignment between multiplex and longer PCR products were shown in Figs. S4–S9. Beta-actin melt-curve and amplicon sequences were shown in Fig. 13, Fig. 14, Fig. 15 and Table S2. The melt-curve of mitochondrial genes of different shrimp species and their respective amplicon sequences were shown in Fig. 1, Fig. 2, Fig. 3, Fig. 4, Fig. 5, Fig. 6, Fig. 7, Fig. 8, Fig. 9, Fig. 10, Fig. 11, Fig. 12 and Table S4–S8. Gene-specific conserved real-time primer and their annealing temperature were shown in Table 3. Species specific mean melting temperature of different mitochondrial genes were shown in Table 4.

**Table 1:** Gene-specific conserved primer and their annealing temperature.

Gene specific conserved primer	Annealing Temperature	Annealing Time
12SrRNA [Crust-12Sf (F) - Crust-12Sr (R)]	55 °C	15 s
16SrRNA [16SAR (F) - 16SBR (R)]	60 °C	
COX1 [COX1F – COX1R; COX1F1 – COX1R1]	55 °C	
Cytb [UCYTB151F - UCYTB270R; UCYTB144F - UCYTB272R]	55 °C	
ND1 [ND1F1/2 – ND1R]	50 or 55 °C

**Table 2:** Beta-actin conserved primers and their annealing temperature.

Primer Name	Sequence (5′-3′)^a^	Combination in PCR (Forward - Reverse)	Annealing Temperature	Annealing Time
B-act FS	AARKSHGGHTTYGCNGGDGAYGA	FM - RS	55 or 60 °C	1 min
B-act FM	GGAYGAYATGGARAAGATYTGGYA	FM - RM		
B-act FE	GARATYGTBCGHGAYRTYAARGA	FM - RE		
B-act RS	TGDCCRTCRGGVAGYTCRTAN	FE - RS		
B-act RM	GGHGGNGCRATGATCTTG	FE - RM		
B-act RE	ATNKNYTGGAAGGTRGANAGVAGA	FE - RE		

a N = A|C|G|T; R = A|G; S = C|G; Y = C|T; K = G|T; D = A|G|T; H = A|C|T; V=A|C|G.

**Table 3:** Gene-specific real-time conserved primers and their annealing temperature.

Gene	Primer Pair	Annealing Temperature
12SrRNA	Crust-12Sf (F) - RT-12Sr	50 or 55 °C
16SrRNA	RT16Sf(N) - RT16Sr	55 or 60 °C
Cytb	RTCytbF1 – RTCytbR1	55 or 60 °C
ND1	RTND1F(N) – RTND1R	50° or 55 °C
COX1	RTCOX2F(M) – RTCOX2R(M)	55 °C
	RTCOXIF(N) – RTCOX1R	
	RTCOX2F(N) – RTCOX2R(N)	
	RTCOXIF(M) - RTCOX1R

**Table 4:** The gene-specific mean melting temperature (T_m_ ± S.D.) value of ten different shrimp species.

Shrimp Species	Lowest observed T_m_ value (˚C)	Highest observed T_m_ value (˚C)	Mean ± S.D.
16SrRNA Gene [RT16Sf(N) - RT16Sr]			
*M. rosenbergii*	76.52	79.49	77.53 ± 0.96
	88.2	88.58	88.37 ± 0.12
*M. japonicus*	74.42	75.32	74.89 ± 0.32
	84.5	88.49	86.87 ± 2.1
*L. vannamei*	71.58	76.8	74.71 ± 2.17
	88.43	88.59	88.51 ± 0.09
*S. crassicornis*	73.42	74.75	74.01 ± 0.44
	84.35	88.51	85.17 ± 1.64
COX1 Gene [RTCOX2F(M) - RTCOX2R(M)]			
*P. uncta*	80.69	81.28	80.96 ± 0.22
*P. hardwickii*	77.55	78.6	78.06 ± 0.39
*L. vannamei*	78.75	79.5	79.2 ± 0.24
*M. rosenbergii*	77.11	77.41	77.26 ± 0.15
*P. monodon*	76.6	77.84	77.26 ± 0.44
Cytb Gene [RTCytbF1 – RTCytbR1]			
*F. merguiensis*	79.04	80.24	79.62 ± 0.51
COX1 Gene [RTCOX2F(M) - RTCOX2R(N)]			
*P. monodon*	76.84	77.49	77.26 ± 0.24
COX1 Gene [RTCOX2F(N) - RTCOX2R(N)]			
*P. monodon*	76.21	78.08	77.03 ± 0.5
*S. crassicornis*	76.81	78.08	77.54 ± 0.42
*P. hardwickii*	77.25	77.7	77.40 ± 0.26
*P. uncta*	80.38	80.54	80.49 ± 0.08
*M. monoceros*	77.7	78.45	78.1 ± 0.38
*M. ensis*	76.8	77.25	77.04 ± 0.17
*L. vannamei*	78.15	78.45	78.35 ± 0.17
*M. rosenbergii*	76.5	76.95	76.73 ± 0.16
*F. merguiensis*	78.3	78.59	78.44 ± 0.14
*M. japonicus*	76.95	77.55	77.2 ± 0.21
COX1 Gene [RTCOX1F(N) - RTCOX1R]			
*P. monodon*	77.55	77.85	77.70 ± 0.12
*P. hardwickii*	78	78.3	78.15 ± 0.15
*P. uncta*	79.64	80.09	79.86 ± 0.19
*M. monoceros*	78.45	78.75	78.6 ± 0.15
*M. ensis*	76.96	77.85	77.49 ± 0.31
*L. vannamei*	77.1	80.53	79 ± 1.64
*M. rosenbergii*	77.69	78.59	78.11 ± 0.39
*F. merguiensis*	77.99	78.59	78.34 ± 0.31
*M. japonicus*	78.29	79.09	78.79 ± 0.28
*S. crassicornis*	78.29	80.53	79.44 ± 0.7
ND1 Gene [RTND1F(N) – RTND1R]			
*M. japonicus*	76.81	77.55	77.23 ± 0.25
*P. uncta*	75.47	76.21	75.79 ± 0.29
COX1 Gene [RTCOX1F(M) - RTCOX1R]			
*P. monodon*	77.4	77.99	77.74 ± 0.2
*P. hardwickii*	77.1	79.04	78.19 ± 0.75
*P. uncta*	77.69	78.59	78.11 ± 0.39
*M. monoceros*	77.99	78.59	78.34 ± 0.31
*M. ensis*	78.29	79.04	78.76 ± 0.26
*L. vannamei*	78.59	79.79	79.25 ± 0.63
*M. rosenbergii*	78.29	80.38	79.56 ± 0.81
*F. merguiensis*	79.33	80.23	79.88 ± 0.48
*M. japonicus*	78.59	80.53	79.37 ± 0.83
*S. crassicornis*	77.4	78.74	78.01 ± 0.44

## 2 Experimental design, materials, and methods

Genomic DNA of ten different shrimp species (*Penaeus monodon*, *Metapenaeus monoceros*, *Metapenaeus ensis*, *Litopenaeus vannamei* or *Penaeus vannamei*, *Parapenaeopsis hardwickii*, *Fenneropenaeus merguiensis* or *Penaeus merguiensis*, *Marsupenaeus japonicus* or *Penaeus japonicus*, *Macrobrachium rosenbergii*, *Solenocera crassicornis*, and *Parapenaeopsis uncta* or *Ganjampenaeopsis uncta*) was analysed by longer PCR or longer-extension PCR method. The modified thermal profile for longer PCR was as follows: 95 °C for 5 min, followed by 40 cycles of 95 °C for 1 min, an experimental annealing temperature of different conserved primers (Table 1; Integrated DNA Technologies, Inc., USA), an extension for 2 min at 72 °C and the final extension at 72 °C for 10 min. The amplified DNA was electrophoresed in 2.0% agarose gel at 90 V for 2 h, and DNA was subsequently stained with ethidium bromide (10 mg mL^−1^) and photographed in a Bio-Rad Molecular Imager® ChemiDoc™ XRS + Imaging System (Bio-Rad Laboratories, Inc., USA). The ND1 gene fragments of *M. japonicus* and *P. uncta* were separated and eluted from 2.0% agarose gel using the commercially available GeneJET Gel Extraction Kit (Thermo Scientific, Massachusetts, USA). The individual eluted DNA fragments were cloned into pTZ57R/T plasmid vector using an InsTAclone PCR Cloning Kit (Thermo Scientific, Massachusetts, USA) and subsequently transformed into *E. coli* XL10 Gold (Thermo Scientific, Massachusetts, USA). The individual plasmid DNA was extracted [3] and sequencing of purified plasmids was performed using BigDye® Terminator v. 3.1 Cycle Sequencing Kit (Applied Biosystems, California, USA) followed by the manufacturer protocol in an automated DNA sequencing machine (Applied Biosystems, California, USA).

Genomic DNA of *P. hardwickii*, and *P. uncta* was analysed by the multiplex PCR method. The modified thermal profile for multiplex PCR was as follows: 94 °C for 6 min, followed by 35 cycles of 94 °C for 70 s, experimental annealing temperature: 50–60 °C for 70 s, an extension at 72 °C for 1 min and the final extension at 72 °C for 15 min. The amplified DNA was electrophoresed in a 2.0% agarose gel at 90 V for 2 h, and eluted from 2.0% agarose gel using the commercially available GeneJET Gel Extraction Kit (Thermo Scientific, Massachusetts, USA). The individual eluted DNA fragments were cloned into pTZ57R/T plasmid vector using an InsTAclone PCR Cloning Kit (Thermo Scientific, Massachusetts, USA) and subsequently transformed into *E. coli* XL10 Gold (Thermo Scientific, Massachusetts, USA). The individual plasmid DNA was extracted [3] and sequencing of purified plasmids was performed using BigDye® Terminator v. 3.1 Cycle Sequencing Kit (Applied Biosystems, California, USA) followed by the manufacturer protocol in an automated DNA sequencing machine (Applied Biosystems, California, USA).

The beta-actin gene sequence of ten different shrimp species was evaluated in this article. The PCR was performed in a thermal cycler (Applied Biosystems, Veriti™ 96 Well Thermal Cycler #9902, California, USA) with 25 μL reaction mixture containing 400 ng genomic DNA of each shrimp species, 200 μM deoxyribonucleotide triphosphates (dNTPs), 2.0 mM MgCl_2_, 1X buffer, and 1.0 U *Taq* DNA polymerase (MP Biomedical, California, USA) and 30 pmole of each oligonucleotide primer (Table 2; Forward – Reverse) from Integrated DNA Technologies (Integrated DNA Technologies, Inc., Iowa, USA). The thermal profile for beta-actin gene was as follows: 94 °C for 5 min, followed by 30 cycles of 94 °C for 45 s, an experimental annealing temperature in Table 2, an extension for 1 min at 72 °C and the final extension at 72 °C for 10 min. The amplified DNA was electrophoresed in 2.0–2.5% agarose gel at 90 V for 2 h, and DNA was subsequently stained with ethidium bromide (10 mg mL^−1^) and photographed in a Bio-Rad Molecular Imager® ChemiDoc™ XRS+ Imaging System (Bio-Rad Laboratories, Inc., USA). The beta-actin gene fragments of ten different shrimp species were separated and eluted from 2.5% agarose gel (Fig. S2A–C) using the commercially available GeneJET Gel Extraction Kit (Thermo Scientific, Massachusetts, USA). The individual eluted DNA fragments were cloned into pTZ57R/T plasmid vector using an InsTAclone PCR Cloning Kit (Thermo Scientific, Massachusetts, USA) and subsequently transformed into *E. coli* XL10 Gold (Thermo Scientific, Massachusetts, USA). The individual plasmid DNA was extracted [3] and sequencing of purified plasmids was performed using BigDye® Terminator v. 3.1 Cycle Sequencing Kit (Applied Biosystems, California, USA) followed by the manufacturer protocol in an automated DNA sequencing machine (Applied Biosystems, California, USA). Sequencing of purified plasmids was performed through the well-known service of SciGenom Labs Pvt. Ltd. (Kochi, India) as well as Central Sanger sequencing facility of Bose Institute.

The real-time PCR was performed in a thermal cycler (StepOnePlus™, Applied Biosystems, USA) with 10–20 μl reaction mixture containing 1–2 μL genomic or plasmid DNA, 2–5 pmol of both gene-specific real-time conserved primers (Table 3; Integrated DNA Technologies, Inc., USA) and 0.8X SYBR® green master mix (Applied Biosystems, USA). The thermal profile was as follows: Holding Stage: 50 °C for 2 mins, 95 °C for 10 min followed by the Cycling Stage: 40 cycles of 95 °C for 30 s, 55–60 °C for 60 s, 72 °C for 2 min, and which was followed by the Melt Curve Stage: 1 min at 95 °C, 2 min at 60 °C, and ended with a denaturation step at 95 °C for 1 min. Melt curve analysis was used to determine the species-specific mean melting temperature (T_m_) of five mitochondrial genes. Species-specific mean melting temperature (T_m_) of four mitochondrial genes were shown in Table 4.
