# Supplementary material for: Extended protocol and real-time PCR dataset for shrimp species identification
Source: Data Brief. 2019 Dec 31;28:105068. doi: 10.1016/j.dib.2019.105068 (PMC6953599; doi:10.1016/j.dib.2019.105068)
Supplement: Multimedia component 1 [file mmc1.pdf]

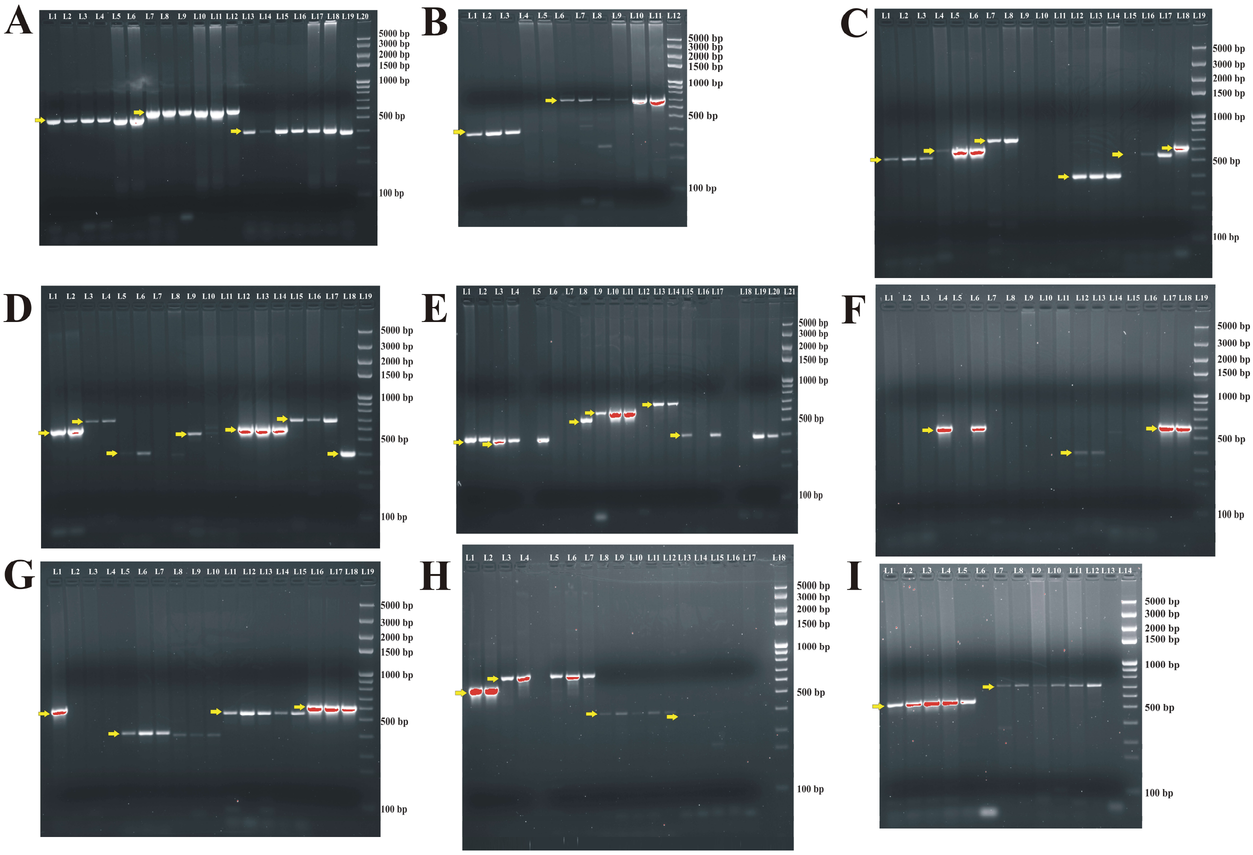

Fig. S1. Longer PCR. A. L1-L2: 16SrRNA gene of *L. vannamei*, L3-L4: 16SrRNA gene of *M. monoceros*, L5-L6: 16SrRNA gene of *M. rosenbergii*, L7-L8: 12SrRNA gene of *L. vannamei*, L9-L10: 12SrRNA gene of *M. monoceros*, L11-L12: 12SrRNA gene of *M. rosenbergii*, L13-L14: Cytb gene of *L. vannamei*, L15-L16: Cytb gene of *M. monoceros*, L17-L18: Cytb gene of *M. rosenbergii*, L19: ND1 gene of *L. vannamei*, L20: a molecular weight marker. **B**. L1: ND1 gene of *L. vannamei*, L2-L3: ND1 gene of *M. monoceros*, L4-L5: ND1 gene of *M. rosenbergii*, L6-L7: COX1 gene of *L. vannamei*, L8-L9: COX1 gene of *M. monoceros*, L10-L11: COX1 gene of *M. rosenbergii*, L12: a molecular weight marker. **C**. L1-L3: 16SrRNA gene of *P. hardwickii*, L4-L6: 12SrRNA gene of *P. hardwickii*, L7-L8: COX1 gene of *P. hardwickii*, L9-L11: Cytb gene of *P. hardwickii*, L12-L14: ND1 gene of *P. hardwickii*, L15-L17: 16SrRNA gene of *P. uncta*, L18: 12SrRNA gene of *P. uncta*, L19: a molecular weight marker. **D**. L1-L2: 12SrRNA gene of *P. uncta*, L3-L4: COX1 gene of *P. uncta*, L5-L6: Cytb gene of *P. uncta*, L7-L8: ND1 gene of *P. uncta*, L9-L11: 16SrRNA gene of *S. crassicornis*, L12-L14: 12SrRNA gene of *S. crassicornis*, L15-L17: COX1 gene of *S. crassicornis*, L18: Cytb gene of *S. crassicornis*, L19: a molecular weight marker. **E**. L1-L2: Cytb gene of *S. crassicornis*, L3-L5: ND1 gene of *S. crassicornis*, L6-L8: 16SrRNA gene of *M. ensis*, L9-L11: 12SrRNA gene of *M. ensis*, L12-L14: COX1 gene of *M. ensis*, L15-L17: Cytb gene of *M. ensis*, L18-L20: ND1 gene of *M. ensis*, L21: a molecular weight marker. **F**. L1-L3: 16SrRNA gene of *F. merguiensis*, L4-L6: 12SrRNA gene of *F. merguiensis*, L7-L8: COX1 gene of *F. merguiensis*, L9-L11: Cytb gene of *F. merguiensis*, L12-L13: ND1 gene of *F. merguiensis*, L14-L16: 16SrRNA gene of *M. japonicus*, L17-L18: 12SrRNA gene of *M. japonicus*, L19: a molecular weight marker. **G**. L1: 12SrRNA gene of *M. japonicus*, L2-L4: COX1 gene of *M. japonicus*, L5-L7: Cytb gene of *M. japonicus*, L8-L10: ND1 gene of *M. japonicus*, L11-L15: 16SrRNA gene of *P. monodon*, L16-L18: 12SrRNA gene of *P. monodon*, L19: a molecular weight marker. **H**. L1-L2: 12SrRNA gene of *P. monodon*, L3-L7: COX1 gene of *P. monodon*, L8-L12: Cytb gene of *P. monodon*, L13-L17: ND1 gene of *P. monodon*, L18: a molecular weight marker. **I**. L1-L5: 16SrRNA gene of *M. japonicus*, L6: negative control, L7-L12: Cytb gene of *M. japonicus*, L13: negative control, L14: a molecular weight marker

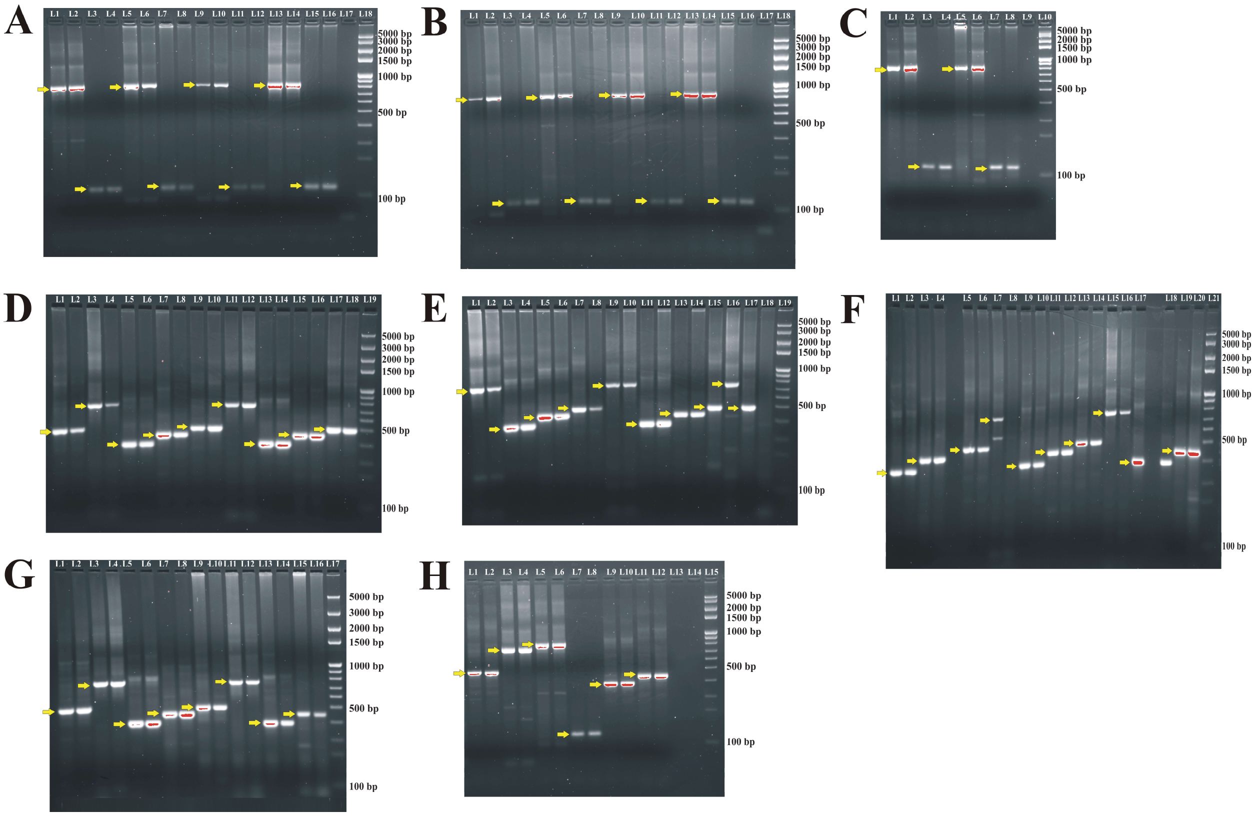

Fig. S2. PCR amplified fragments of Beta-actin gene. **A**. L1-L2: Beta-actin gene of *P. monodon* amplified by B-act FM – B-act RE primer, L3-L4: Beta-actin gene of *P. monodon* amplified by B-act FE – B-act RS primer, L5-L6: Beta-actin gene of *P. hardwickii* amplified by B-act FM – B-act RE primer, L7-L8: Beta-actin gene of *P. hardwickii* amplified by B-act FE – B-act RS primer, L9-L10: Beta-actin gene of *P. uncta* amplified by B-act FM – B-act RE primer, L11-L12: Beta-actin gene of *P. uncta* amplified by B-act FE – B-act RS primer, L13-L14: Beta-actin gene of *S. crassicornis* amplified by B-act FM – B-act RE primer, L15-L16: Beta-actin gene of *S. crassicornis* amplified by B-act FE – B-act RS primer, L17: Negative Control, L18: a molecular weight marker. **B**. L1-L2: Beta-actin gene of *M. ensis* amplified by B-act FM – B-act RE primer, L3-L4: Beta-actin gene of *M. ensis* amplified by B-act FE – B-act RS primer, L5-L6: Beta-actin gene of *F. merguiensis* amplified by B-act FM – B-act RE primer, L7-L8: Beta-actin gene of *F. merguiensis* amplified by B-act FE – B-act RS primer, L9-L10: Beta-actin gene of *M. japonicus* amplified by B-act FM – B-act RE primer, L11-L12: Beta-actin gene of *M. japonicus* amplified by B-act FE – B-act RS primer, L13-L14: Beta-actin gene of *L. vannamei* amplified by B-act FM – B-act RE primer, L15-L16: Beta-actin gene of *L. vannamei* amplified by B-act FE – B-act RS primer, L17: Negative Control, L18: a molecular weight marker. **C**. L1-L2: Beta-actin gene of *M. monoceros* amplified by B-act FM – B-act RE primer, L3-L4: Beta-actin gene of *M. monoceros* amplified by B-act FE – B-act RS primer, L5-L6: Beta-actin gene of *M. rosenbergii* amplified by B-act FM – B-act RE primer, L7-L8: Beta-actin gene of *M. rosenbergii* amplified by B-act FE – B-act RS primer, L9: Negative Control, L10: a molecular weight marker. **D**. L1-L2: Beta-actin gene of *P. hardwickii* amplified by B-act FM – B-act RS primer, L3-L4: Beta-actin gene of *P. hardwickii* amplified by B-act FM – B-act RM primer, L5-L6: Beta-actin gene of *P. hardwickii* amplified by B-act FE – B-act RM primer, L7-L8: Beta-actin gene of *P. hardwickii* amplified by B-act FE – B-act RE primer, L9-L10: Beta-actin gene of *P. uncta* amplified by B-act FM – B-act RS primer, L11-L12: Beta-actin gene of *P. uncta* amplified by B-act FM – B-act RM primer, L13-L14: Beta-actin gene of *P. uncta* amplified by B-act FE – B-act RM primer, L15-L16: Beta-actin gene of *P. uncta* amplified by B-act FE – B-act RE primer, L17-L18: Beta-actin gene of *S. crassicornis* amplified by B-act FM – B-act RS primer, L18: a molecular weight marker. **E**. L1-L2: Beta-actin gene of *S. crassicornis* amplified by B-act FM – B-act RM primer, L3-L4: Beta-actin gene of *S. crassicornis* amplified by B-act FE – B-act RM primer, L5-L6:

Beta-actin gene of *S. crassicornis* amplified by B-act FE – B-act RE primer, L7-L8: Beta-actin gene of *M. ensis* amplified by B-act FM – B-act RS primer, L9-L10: Beta-actin gene of *M. ensis* amplified by B-act FM – B-act RM primer, L11-L12: Beta-actin gene of *M. ensis* amplified by B-act FE – B-act RM primer, L13-L14: Beta-actin gene of *M. ensis* amplified by B-act FE – B-act RE primer, L15: Beta-actin gene of *F. merguiensis* amplified by B-act FM – B-act RS primer, L16: Beta-actin gene of *F. merguiensis* amplified by B-act FM – B-act RM primer, L17: Beta-actin gene of *F. merguiensis* amplified by B-act FM – B-act RS primer, L18: Beta-actin gene of *F. merguiensis* amplified by B-act FM – B-act RM primer, L19: a molecular weight marker. **F**. L1-L2: Beta-actin gene of *F. merguiensis* amplified by B-act FE – B-act RM primer, L3-L4: Beta-actin gene of *F. merguiensis* amplified by B-act FE – B-act RE primer, L5-L6: Beta-actin gene of *M. japonicus* amplified by B-act FM – B-act RS primer, L7-L8: Beta-actin gene of M. japonicus amplified by B-act FM – B-act RM primer, L9-L10: Beta-actin gene of *M. japonicus* amplified by B-act FE – B-act RM primer, L11-L12: Beta-actin gene of *M. japonicus* amplified by B-act FE – B-act RE primer, L13-L14: Beta-actin gene of *L. vannamei* amplified by B-act FM – B-act RS primer, L15-L16: Beta-actin gene of *L. vannamei* amplified by B-act FM – B-act RM primer, L17-L18: Beta-actin gene of *L. vannamei* amplified by B-act FE – B-act RM primer, L19-L20: Beta-actin gene of *L. vannamei* amplified by B-act FE – B-act RE primer, L21: a molecular weight marker. **F**. L1-L2: Beta-actin gene of *M. monoceros* amplified by B-act FM – B-act RS primer, L3-L4: Beta-actin gene of *M. monoceros* amplified by B-act FM – B-act RM primer, L5-L6: Beta-actin gene of *M. monoceros* amplified by B-act FE – B-act RM primer, L7-L8: Beta-actin gene of *M. monoceros* amplified by B-act FE – B-act RE primer, L9-L10: Beta-actin gene of *M. rosenbergii* amplified by B-act FM – B-act RS primer, L11-L12: Beta-actin gene of *M. rosenbergii* amplified by B-act FM – B-act RM primer, L13-L14: Beta-actin gene of *M. rosenbergii* amplified by B-act FE – B-act RM primer, L15-L16: Beta-actin gene of *M. rosenbergii* amplified by B-act FE – B-act RE primer, L17: a molecular weight marker. G. L1-L12: PCR amplified fragments from *P. monodon* using above-mentioned beta-actin primers, L15: a molecular weight marker.

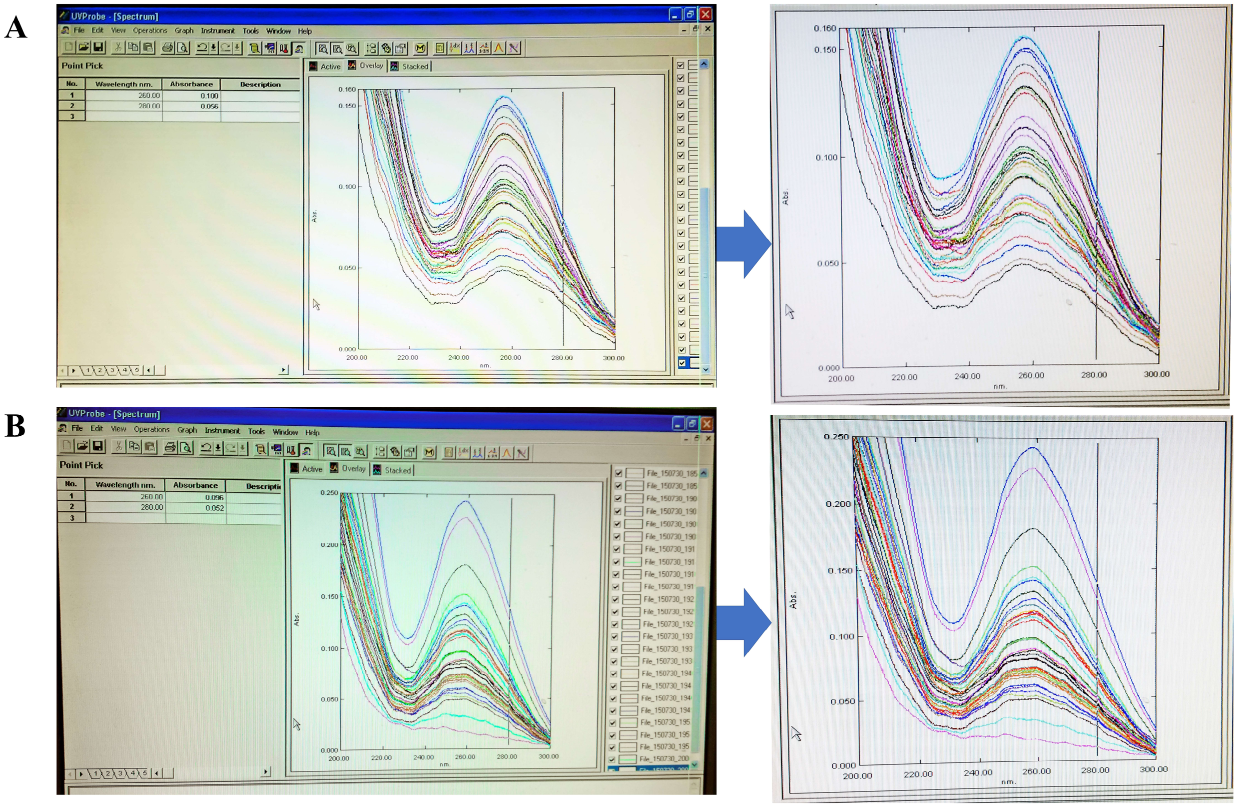

Fig. S3. UV-absorbance Spectrum of purified plasmids. Quality of the purified cloned plasmids was determined by spectrophotometry using a Shimadzu UV160U spectrophotometer (Shimadzu Corp., Japan).

```
                   *        20         *        40         *        60         *        80         *       100         *       120
Multiplex : CAGCAGTCGCGGTTATACTGAGAGGTAAAATAAAAGATAATTGGTTAAAAATGCTTAAATGTAAAATTTTATTTGAACGGATTGTTTTGTGTTAATAAAGGGTGAAATCAATTTTTTATTATATATAAT : 127
Longer    : CAGCAGTCGCGGTTATACTGAGAGGTAAAATAAAAGATAATTGGTTAAAAATGCTTAAATGTAAAATTTTATTTGAACGGATTGTTTTGTGTTAATAAAGGGTGAAATCAATTTTTTATTATATATAAT : 127
            CAGCAGTCGCGGTTATACTGAGAGGTAAAATAAAAGATAATTGGTTAAAAATGCTTAAATGTAAAATTTTATTTGAACGGATTGTTTTGTGTTAATAAAGGGTGAAATCAATTTTTTATTATATATAAT

              *       140         *       160         *       180         *       200         *       220         *       240         *
Multiplex : TAATTCTGTTAAATGAGAGTTGTTGAGGTTGTTAAGGTTAAGGTATGAACCAGGATTAGATACCCTGTTACACTTTACTTAAAAATTTATTACACCTAGGTAGTAAGCAGTTATGATCTTGAAACTT : 254
Longer    : TAATTCTGTTAAATGAGAGTTGTTGAGGTTGTTAAGGTTAAGGTATGAACCAGGATTAGATACCCTGTTACACTTTACTTAAAAATTTATTACACCTAGGTAGTAAGCAGTTATGATCTTGAAACTT : 254
            TAATTCTGTTAAATGAGAGTTGTTGAGGTTGTTAAGGTTAAGGTATGAACCAGGATTAGATACCCTGTTACACTTTACTTAAAAATTTATTACACCTAGGTAGTAAGCAGTTATGATCTTGAAACTT

               260         *       280         *       300         *       320         *       340         *       360         *       380
Multiplex : AAAGGATTTGGCGGTAATTTAGTCTAGTTAGAGGAACCTGTCCTGTAATCGATAAACCACGTAGTATCTTACTTTATCTTGAATTTTTCAGTTTATATACCATCATTGTTAGATAACTTTAAGAAAA : 381
Longer    : AAAGGATTTGGCGGTAATTTAGTCTAGTTAGAGGAACCTGTCCTGTAATCGATAAACCACGTAGTATCTTACTTTATCTTGAATTTTTCAGTTTATATACCATCATTGTTAGATAACTTTAAGAAAA : 381
            AAAGGATTTGGCGGTAATTTAGTCTAGTTAGAGGAACCTGTCCTGTAATCGATAAACCACGTAGTATCTTACTTTATCTTGAATTTTTCAGTTTATATACCATCATTGTTAGATAACTTTAAGAAAA

                    *       400         *       420         *       440         *       460         *       480         *       500
Multiplex : TTAAGAAGTTATTAAAATAATTTTAAGTTAATATATTAGATCAAGGTGTAGCTAATGATAAAGTAGAGATGGGTTACAATAATATTAGTTTATAACGGATTTAAAAAAGAATTTTTTAAAGAAGGAG : 508
Longer    : TTAAGAAGTTATTAAAATAATTTTAAGTTAATATATTAGATCAAGGTGTAGCTAATGATAAAGTAGAGATGGGTTACAATAATATTAGTTTATAACGGATTTAAAAAAGAATTTTTTAAAGAAGGAG : 508
            TTAAGAAGTTATTAAAATAATTTTAAGTTAATATATTAGATCAAGGTGTAGCTAATGATAAAGTAGAGATGGGTTACAATAATATTAGTTTATAACGGATTTAAAAAAGAATTTTTTAAAGAAGGAG

              *       520         *       540         *       560         *       580         *       600         *       620
Multiplex : GATTTAAATGTAAATGTGTTTTAATATGGCATATTGATTATAGCTCTAGATTATGTACACATCGCCCGTCGCTCTCGTTATATAAAGCGAGATAAGTCGTAACAAAGTAGGTGT : 622
Longer    : GATTTAAATGTAAATGTGTTTTAATATGGCATATTGATTATAGCTCTAGATTATGTACACATCGCCCGTCGCTCTCGTTATATAAAGCGAGATAAGTCGTAACAAAGTAGGTGT : 622
            GATTTAAATGTAAATGTGTTTTAATATGGCATATTGATTATAGCTCTAGATTATGTACACATCGCCCGTCGCTCTCGTTATATAAAGCGAGATAAGTCGTAACAAAGTAGGTGT
```

Fig. S4. Pairwise sequence alignment of 12SrRNA gene which was amplified by multiplex PCR (multiple primer pair with single template) and longer PCR (single primer pair with single template) method. The 12SrRNA gene sequence was amplified from genomic DNA of *P. hardwickii*.

```
                    *        20         *        40         *        60         *        80         *       100         *       120
Multiplex : CAGCAGCCGCGGTTATACTGAGAGGTAAAGTAAAAGATAGTCGGTTAAAAATGCTTAGATGAAAAGATTTTATTTAAATGGATTGTTTTGTGTTAATAAAGGGTGAAATCGATTTTTTGCTATATAA : 127
Longer    : CAGCAGTCGCGGTTATACTGAGAGGTAAAGTAAAAGATAGTCGGTTAAAAATGCTTAGATGAAAAGATTTTATTTAAATGGATTGTTTTGTGTTAATAAAGGGTGAAATCGATTTTTTGCTATATAA : 127
            CAGCAG CGCGGTTATACTGAGAGGTAAAGTAAAAGATAGTCGGTTAAAAATGCTTAGATGAAAAGATTTTATTTAAATGGATTGTTTTGTGTTAATAAAGGGTGAAATCGATTTTTTGCTATATAA

              *       140         *       160         *       180         *       200         *       220         *       240         *
Multiplex : TTAATTTCGTTAGATAAAAGTTGTTGAGGTTATTAAGGTTAAGGTATGAACCAGGATTAGATACCCTGTTACACTTTACTTAAAAAGTTTATTATACCCAGGTAATAAGCAGTTATGATCTTGAAAC : 254
Longer    : TTAATTTCGTTAGATAAAAGTTGTTGAGGTTATTAAGGTTAAGGTATGAACCAGGATTAGATACCCTGTTACACTTTACTTAAAAAGTTTATTATACCCAGGTAATAAGCAGTTATGATCTTGAAAC : 254
            TTAATTTCGTTAGATAAAAGTTGTTGAGGTTATTAAGGTTAAGGTATGAACCAGGATTAGATACCCTGTTACACTTTACTTAAAAAGTTTATTATACCCAGGTAATAAGCAGTTATGATCTTGAAAC

               260         *       280         *       300         *       320         *       340         *       360         *       380
Multiplex : TTAAAGGATTTGGCGGTAATTTAGTCTAGTTAGAGGAACCTGTCCTGTAATCGATAAACCACGCAGTATCTTACTTTATCTTGAAAATTTATCAGTTTATATACCATCATTATTAGATAACTTTAAA : 381
Longer    : TTAAAGGATTTGGCGGTAATTTAGTCTAGTTAGAGGAACCTGTCCTGTAATCGATAAACCACGCAGTATCTTACTTTATCTTGAAAATTTATCAGTTTATATACCATCATTATTAGATAACTTTAAA : 381
            TTAAAGGATTTGGCGGTAATTTAGTCTAGTTAGAGGAACCTGTCCTGTAATCGATAAACCACGCAGTATCTTACTTTATCTTGAAAATTTATCAGTTTATATACCATCATTATTAGATAACTTTAAA

                 *       400         *       420         *       440         *       460         *       480         *       500
Multiplex : AAGGTAGAGAAGTTATTGAAATAATTTTAAGTTAGTATATTAGATCAAGGTGTAGCTAATGATAAAGTAGAGATGGGTTACAATAATATAAGTTTATAACGGATTTAAAGGGGAATTTTTTTTAATG : 508
Longer    : AAGGTAGAGAAGTTATTGAAATAATTTTAAGTTAGTATATTAGATCAAGGTGTAGCTAATGATAAAGTAGAGATGGGTTACAATAATATAAGTTTATAACGGATTTAAAGGGGAATTTTTTTTAATG : 508
            AAGGTAGAGAAGTTATTGAAATAATTTTAAGTTAGTATATTAGATCAAGGTGTAGCTAATGATAAAGTAGAGATGGGTTACAATAATATAAGTTTATAACGGATTTAAAGGGGAATTTTTTTTAATG

             *       520         *       540         *       560         *       580         *       600         *       620
Multiplex : AAGGAGGATTTAAATGTAAGTATATTTTAACATGATATACTGATTATAGCTCTAGATTATGTACACATCGCCCGTCGCTCTCGTTATATAAAGCGAGATAAGTCGTAACAAAGCAGGTGT : 628
Longer    : AAGGAGGATTTAAATGTAAGTATATTTTAACATGATATACTGATTATAGCTCTAGATTATGTACACATCGCCCGTCGCTCTCGTTATATAAAGCGAGATAAGTCGTAACAAAGTAGGTGT : 628
            AAGGAGGATTTAAATGTAAGTATATTTTAACATGATATACTGATTATAGCTCTAGATTATGTACACATCGCCCGTCGCTCTCGTTATATAAAGCGAGATAAGTCGTAACAAAG AGGTGT
```

Fig. S5. Pairwise sequence alignment of 12SrRNA gene which was amplified by multiplex PCR (multiple primer pair with single template) and longer PCR (single primer pair with single template) method. The 12SrRNA gene sequence was amplified from genomic DNA of *P. uncta*.

```
                     *        20         *        40         *        60         *        80         *       100         *       120
Multiplex : CGCCTGTTTATCAAAAACATGTCTGTATGATGGTTATATAGAGTCTGGCCTGCCCACTGATTAAGGTTAAAGGGCCGCGGTATTTTGACCGTGCGAAGGTAGCATAATCATTAGTCTTTTAATTGAG : 127
Longer    : CGCCTGTTTATCAAAAACATGTCTGTATGATGGTTATATAGAGTCTGGCCTGCCCACTGATTAAGGTTAAAGGGCCGCGGTATTTTGACCGTGCGAAGGTAGCATAATCATTAGTCTTTTAATTGAG : 127
            CGCCTGTTTATCAAAAACATGTCTGTATGATGGTTATATAGAGTCTGGCCTGCCCACTGATTAAGGTTAAAGGGCCGCGGTATTTTGACCGTGCGAAGGTAGCATAATCATTAGTCTTTTAATTGAG

              *       140         *       160         *       180         *       200         *       220         *       240         *
Multiplex : GGCTTGTATGAATGGTTGAACAAAAAAATAAGCTGTCTCGGTTATAAAAAATTGAATTTAACTTTTAAGTGAAAAGGCTTAAATGATTTAGGATGACGATAAGACCCTATAAAGCTTTACAATTATTTG : 254
Longer    : GGCTTGTATGAATGGTTGAACAAAAAAATAAGCTGTCTCGGTTATAAAAAATTGAATTTAACTTTTAAGTGAAAAGGCTTAAATGATTTAGGATGACGATAAGACCCTATAAAGCTTTACAATTATTTG : 254
            GGCTTGTATGAATGGTTGAACAAAAAAATAAGCTGTCTCGGTTATAAAAAATTGAATTTAACTTTTAAGTGAAAAGGCTTAAATGATTTAGGATGACGATAAGACCCTATAAAGCTTTACAATTATTTG

               260         *       280         *       300         *       320         *       340         *       360         *       380
Multiplex : ATTGAATTATAAATTGTTAGTATAAATTTAGTTTAATTAATGTTTGTTTCGTTGGGGCGACGGGAATATAATATTGTAACTGTTTTATAATTTTATAACAATAATTTTTGGTGAAGATGATCCTTTA : 381
Longer    : ATTGAATTATAAATTGTTAGTATAAATTTAGTCTAATTAATGTTTGTTTCGTTGGGGCGACGGGAATATAATATTGTAACTGTTTTATAATTTTATAACAATAATTTTTGGTGAAGATGATCCTTTA : 381
            ATTGAATTATAAATTGTTAGTATAAATTTAGT TAATTAATGTTTGTTTCGTTGGGGCGACGGGAATATAATATTGTAACTGTTTTATAATTTTATAACAATAATTTTTGGTGAAGATGATCCTTTA

                    *       400         *       420         *       440         *       460         *       480         *       500
Multiplex : TTAGAGATTAAAAGATTAAGTTACTTTAGGGATAACAGCGTAATCTTCTTTAAAAGTTCATATCGACAAGAAGGGTTGCGACCTCGATGTTGAATTAAGGTTTCTTTGTGGTGCAGAAGTTACAATA : 508
Longer    : TTAGAGATTAAAAGATTAAGTTACTTTAGGGATAACAGCGTAATCTTCTTTAAAAGTTCATATCGACAAGAAGGGTTGCGACCTCGATGTTGAATTAAGGTTTCTTTGTGGTGCAGAAGTTACAATA : 508
            TTAGAGATTAAAAGATTAAGTTACTTTAGGGATAACAGCGTAATCTTCTTTAAAAGTTCATATCGACAAGAAGGGTTGCGACCTCGATGTTGAATTAAGGTTTCTTTGTGGTGCAGAAGTTACAATA

             *       520         *       540         *
Multiplex : AGAAGGTCTGTTCGACCTTTAATTCCTTACATGATCTGAGTTCAAACCGG : 558
Longer    : AGAAGGTCTGTTCGACCTTTAATTCCTTACATGATCTGAGTTCAAACCGG : 558
            AGAAGGTCTGTTCGACCTTTAATTCCTTACATGATCTGAGTTCAAACCGG
```

Fig. S6. Pairwise sequence alignment of 16SrRNA gene which was amplified by multiplex PCR (multiple primer pair with single template) and longer PCR (single primer pair with single template) method. The 16SrRNA gene sequence was amplified from genomic DNA of *P. uncta*.

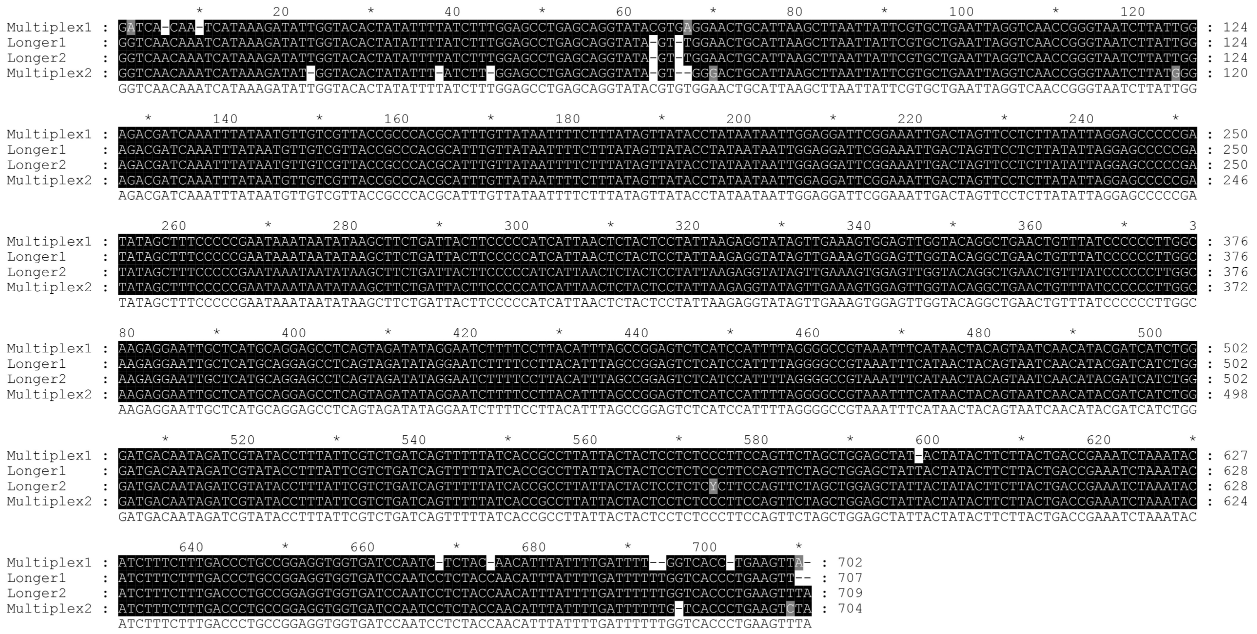

Fig. S7. Pairwise sequence alignment of COX1 gene which was amplified by multiplex PCR (multiple primer pair with single template) and longer PCR (single primer pair with single template) method. The COX1 gene sequence was amplified from genomic DNA of *P. hardwickii*.

```
                     *        20         *        40         *        60         *        80         *       100         *       120
Multiplex : TTTCTACAAATCATAAA-GACATCGGAACCCTATATTTTATCTTCGGGGCTTGAGCAGGAATGGTAGGTACGGCTCTCAGACTAATTATCCGGGCCGAACTCGGTCAACCAGGCAATCTTATTGGAG : 126
Longer    : TTTCTACAAATCATAAAAGACATCGGAACCCTATATTTTATCTTCGGGGCTTGAGCAGGAATGGTAGGTACGGCTCTCAGACTAATTATCCGGGCCGAACTCGGTCAACCAGGCAATCTTATTGGAG : 127
            TTTCTACAAATCATAAA GACATCGGAACCCTATATTTTATCTTCGGGGCTTGAGCAGGAATGGTAGGTACGGCTCTCAGACTAATTATCCGGGCCGAACTCGGTCAACCAGGCAATCTTATTGGAG

              *       140         *       160         *       180         *       200         *       220         *       240         *
Multiplex : ATGACCAAATCTATAATGTTGTGGTCACCGCACATGCTTTTGTAATAATTTTTTTCATGGTTATACCTATGATAATTGGAGGATTTGGAAACTGATTAGTCCCCCTAATACTTGGAGCCCCAGATAT : 253
Longer    : ATGACCAAATCTATAATGTTGTGGTCACCGCACATGCTTTTGTAATAATTTTTTTCATGGTTATACCTATGATAATTGGAGGATTTGGAAACTGATTAGTCCCCCTAATACTTGGAGCCCCAGATAT : 254
            ATGACCAAATCTATAATGTTGTGGTCACCGCACATGCTTTTGTAATAATTTTTTTCATGGTTATACCTATGATAATTGGAGGATTTGGAAACTGATTAGTCCCCCTAATACTTGGAGCCCCAGATAT

               260         *       280         *       300         *       320         *       340         *       360         *       380
Multiplex : AGCTTTCCCACGAATGAATAATATAAGATTCTGGCTCCTTCCTCCATCCCTGACTCTTCTCCTCTCGAGCGGAATAGTAGAAAGAGGGGTAGGGACCGGCTGAACAGTGTACCCTCCCTTAGCTAGA : 380
Longer    : AGCTTTCCCACGAATGAATAATATAAGATTCTGGCTCCTTCCTCCATCCCTGACTCTTCTCCTCTCGAGCGGAATAGTAGAAAGAGGGGTAGGGACCGGCTGAACAGTGTACCCTCCCTTAGCTAGA : 381
            AGCTTTCCCACGAATGAATAATATAAGATTCTGGCTCCTTCCTCCATCCCTGACTCTTCTCCTCTCGAGCGGAATAGTAGAAAGAGGGGTAGGGACCGGCTGAACAGTGTACCCTCCCTTAGCTAGA

                    *       400         *       420         *       440         *       460         *       480         *       500
Multiplex : GGAATCGCACACGCAGGTGCTTCAGTAGATATAGGAATCTTCTCCCTCCACCTGGCAGGGGTCTCATCAATCCTGGGAGCCGTAAACTTTATAACCACAGTAATCAACATACGATCTTCAGGAATAT : 507
Longer    : GGAATCGCACACGCAGGTGCTTCAGTAGATATAGGAATCTTCTCCCTCCACCTGGCAGGGGTCTCATCAATCCTGGGAGCCGTAAACTTTATAACCACAGTAATCAACATACGATCTTCAGGAATAT : 508
            GGAATCGCACACGCAGGTGCTTCAGTAGATATAGGAATCTTCTCCCTCCACCTGGCAGGGGTCTCATCAATCCTGGGAGCCGTAAACTTTATAACCACAGTAATCAACATACGATCTTCAGGAATAT

             *       520         *       540         *       560         *       580         *       600         *       620         *
Multiplex : CGATGGACCGCATACCCTTGTTTGTATGGTCAGTATTCATTACTGCTCTTCTCCTTCTTCTCTCTTTACCAGTCTTAGCAGGGGCTATCACAATACTCTTAACAGACCGTAACTTGAATACATCTTT : 634
Longer    : CGATGGACCGCATACCCTTGTTTGTATGGTCAGTATTCATTACTGCTCTTCTCCTTCTTCTCTCTTTACCAGTCTTAGCAGGGGCTATCACAATACTCTTAACAGACCGTAACTTGAATACATCTTT : 635
            CGATGGACCGCATACCCTTGTTTGTATGGTCAGTATTCATTACTGCTCTTCTCCTTCTTCTCTCTTTACCAGTCTTAGCAGGGGCTATCACAATACTCTTAACAGACCGTAACTTGAATACATCTTT

              640         *       660         *       680         *       700         *
Multiplex : CTTTGACCCGGCCGGAGGAGGGGACCCTATCCTCTATCA-CACTTGTTTTGATTTTTCGGTCATCCTGAAGTATA : 708
Longer    : CTTTGACCCGGCCGGAGGAGGGGACCCTATCCTCTATCAACACTTGTTTTGATTTTTCGGTCATCCTGAAGTATA : 710
            CTTTGACCCGGCCGGAGGAGGGGACCCTATCCTCTATCA CACTTGTTTTGATTTTTCGGTCATCCTGAAGTATA
```

Fig. S8. Pairwise sequence alignment of COX1 gene which was amplified by multiplex PCR (multiple primer pair with single template) and longer PCR (single primer pair with single template) method. The COX1 gene sequence was amplified from genomic DNA of *P. uncta*.

```
                   *        20         *        40         *        60         *        80         *       100         *       120
Multiplex : AAGCTAATCTAACTTCATAAGAAATAGTCTGTGCCACAGCTCGAAGTCTCCCTAACAAAGCATACTTAGAATTAGAAGCTCATCCAGCCCCTATCGTAGTGTATACCCCCAAACTAGTGCAGCAAAG : 127
Longer    : AAGCTAATCTAACTTCATAAGAAATAGTCTGTGCCACAGCTCGAAGTCTCCCTAACAAAGCATACTTAGAATTAGAAGCTCATCCAGCCCCTATCGTAGTGTATACCCCCAAACTAGTGCAGCAAAG : 127
            AAGCTAATCTAACTTCATAAGAAATAGTCTGTGCCACAGCTCGAAGTCTCCCTAACAAAGCATACTTAGAATTAGAAGCTCATCCAGCCCCTATCGTAGTGTATACCCCCAAACTAGTGCAGCAAAG

              *       140         *       160         *       180         *       200         *       220         *       240         *
Multiplex : AAAAAACAATACACCTATCTTAAAATTTATTAAACCTAATTCATAAGGTATCACTAACCAAACAATCAAAGATACTAACAGCCTAAAAACAGGAGATAGATAATAAGGCAAAAAATTAGATATTGTT : 254
Longer    : AAAAAACAATACACCTATCTTAAAATTTATTAAACCTAATTCATAAGGTATCACTAACCAAACAATCAAAGATACTAACAGCCTAAAAACAGGAGATAGATAATAAGGCAAAAAATTAGATATTGTT : 254
            AAAAAACAATACACCTATCTTAAAATTTATTAAACCTAATTCATAAGGTATCACTAACCAAACAATCAAAGATACTAACAGCCTAAAAACAGGAGATAGATAATAAGGCAAAAAATTAGATATTGTT

               260         *       280         *       300         *       320         *       340         *       360         *       380
Multiplex : GGCAAAGTCTGTTCTTTCCTAAATAACTTAACTACATCCGAAAAAGGCTGTAATAACCCCATATACCCAACCTTATTTGGACCCTTACGAATTTGAATATAACCTAAAACTTTTCGTTCTAATAAAG : 381
Longer    : GGCAAAGTCTGTTCTTTCCTAAATAACTTAACTACATCCGAAAAAGGCTGTAATAACCCCATATACCCAACCTTATTTGGACCCTTACGAATTTGAATATAACCTAAAACTTTTCGTTCTAATAAAG : 381
            GGCAAAGTCTGTTCTTTCCTAAATAACTTAACTACATCCGAAAAAGGCTGTAATAACCCCATATACCCAACCTTATTTGGACCCTTACGAATTTGAATATAACCTAAAACTTTTCGTTCTAATAAAG

                   *
Multiplex : TTAAAAATGCTAC : 394
Longer    : TTAAAAATGCTAC : 394
            TTAAAAATGCTAC
```

Fig. S9. Pairwise sequence alignment of ND1 gene which was amplified by multiplex PCR (multiple primer pair with single template) and longer PCR (single primer pair with single template) method. The ND1 gene sequence was amplified from genomic DNA of *P. hardwickii*.

TableS1. The ND1 gene sequences (Longer PCR Product)

Shrimp Species	Gene Name	Primer Set	Nucleotide Sequence	Amino Acid Sequence
M. japonicus (394 bp)	ND1	ND1F1/2 – ND1R (Conserved Primer)	AAGCTAATCTAACTTCATAAGAAATAGTTTGAGCCACCGCTCGAAGTCTCCCTAATAAAGAATATTTAGAAT<br>TAGAAGCTCACCCTGCTCTTATAGTCGTGTATACCCCTAAACTAGTGCAACACAAAAAAAATAAAGTACTTAT<br>TCTAAAATTTATCAAACCCAACTCGTACGGTATTACCAGTCAAACAATTAATGAAACAAACAAACTAAATACTG<br>GTGATAAGTAATAAGGAAGAAAATTAGATATAACAGGAAGAGTCTGCTCTTTCGTGAATAACTTAACAGCATCA<br>GCAAAAGGCTGCAACAACCCTATAAAACCCACTTTATTAGGTCCTTTACGAATTTGAATGTAACCAAGAATCTTA<br>CGTTCTAATAAAGTTAAAAATGCTAC	VAFLTLLERKILGYIQIRKGPNKVGFMGLLQPFADAVKLFTKEQTLPVMSNFLPYYLSPVFSLFVSLIV<br>WLVMPYELGLMNFSMSTLFFLCCTSLGVYTTMSAGWASNSKYSLLGSLRAVAQTISYEVSLA
P. uncta, Clone 1 (394 bp)			AAGCTAATCTAACTTCATAAGAAATAGTTTGAGCTACCGCGCGCAAACTTCCTAGTAAAGCGTATTTAGAATTAGAA<br>GATCATCCAGCTCTTATTGTAGTATATACCCCTAAGCTAGTACAACATAAAAAGAATAAAACTCTCATTCTAAAGTTTA<br>TTAAACCCAACTCATATGGTATAACCAACCATACAATCAAAGAAACAAACAAACTAAATACAGGAGACAAATAATATG<br>GAAGAAAATTAGATATTACAGGAAGAGTTTGCTCCTTTGTAAAAAGTTTAATTGCATCTGCAAATGGCTGTAACAATCCT<br>ATAAACCCCACTTTATTAGGACCCTTACGAATTTGGATATAACCTAAAATCTTTCGTTCTAATAAAGTTAAAAATGCTAC	VAFLTLLERKILGYIQIRKGPNKVGFMGLLQPFADAIKLFTKEQTLPVMSNFLPYYLSPVFSLFVSLIVWL<br>VMPYELGLMNFSMSVLFFLCCTSLGVYTTMSAGWSSNSKYALLGSLRAVAQTISYEVSLA
P. uncta, Clone 2 (394 bp)			AAGCTAATCTAACTTCATAAGAAATAGTCTGAGCTACCGCGCGCAAACTTCCTAGTAAAGCGTATTTAGAATTAGAAGA<br>TCACCCAGCTCTTATTGTAGTATATACCCCTAAGCTAGTACAACATAAAAAGAATAAAACTCTCATTCTAAAGTTTATTA<br>AACCCAACTCATATGGTATAACCAACCATACAATCAAAGAAACAAACAAACTAAATACAGGAGACAAATAATATGGAA<br>GAAAATTAGATATTACAGGAAGAGTTTGCTCCTTTGTAAAAAGTTTAATTGCATCTGCAAATGGCTGTAACAATCCTATA<br>AACCCCACTTTATTAGGACCCTTACGAATTTGGATATAACCTAAAATCTTTCGTTCTAATAAAGTTAAAAATGCTAC	VAFLTLLERKILGYIQIRKGPNKVGFMGLLQPFADAIKLFTKEQTLPVMSNFLPYYLSPVFSLFVSLIV<br>WLVMPYELGLMNFSMSVLFFLCCTSLGVYTTMSAGWSSNSKYALLGSLRAVAQTISYEVSLA

Table S2. The sequence of PCR amplified fragment by conserved beta-actin gene-specific real-time primer pair

Shrimp Species	Gene Name	Primer Set (Real-time)	Nucleotide Sequence
F. merguiensis	Beta-actin	B-act FE – B-act RS	GAGATTGTGCGAGATGTTAAGGAGAAGCTTTGCTACATCGCCCTTGACTTCGAGAGTGAGATGAACGTCGCTGCTGCTTCCTCTTCCCTGGACAAGTCCTACGAACTCCCCGATGGCCA
L. vannamei			GAAATCGTGCGTGACGTCAAGGAGAAGCTTTGCTACGTCGCTCTTGACTTCGAGAGTGAGATGACAATGGCTGCCGCATCGTCTTCCGTTGAGAAGTCGTACGAACTCCCCGATGGACA
M. ensis			GAGATCGTGCGAGATGTCAAGGAGAAGCTTTGCTATGTCGCTCTCGACTTCGAGAGTGAGATGACCATGTCTGCCGCATCCTCGTCAATCGAAAAGTCGTACGAACTTCCCGACGGCCA
M. japonicus			GAGATTGTGCGAGACGTTAAGGAGAAGCTTTGCTACATCGCCCTTGACTTCGAGAGTGAGATGAACGTCGCTGCCGCTTCCTCTTCCTTGGACAAGTCCTACGAGCTGCCCGACGGCCA
M. monoceros			GAGATCGTGCGTGATATCAAGGAAAAGCTTTGCTACATTGCTCTTGACTTCGAGAATGAAATGGCTCAGGCAGCTGCATCCAGCTCTCTGGACAAGTCTTACGAACTTCCCGACGGACA
M. rosenbergii			GAGATTGTGCGTGACGTTAAAGAAAAGCTCTGCTACGTCGCATTGGACTTCGAGCAGGAGATGACCACTGCCGCCTCCTCCTCTTCCCTGGAGAAGTCGTACGAACTTCCCGACGGCCA
P. hardwickii			GAGATCGTGCGCGTGTCAAGGAGAAGCTTTGCTATGTCGCCCTCGATTTCGAGAACGAAATGGCTCAGGCTGCTGCCTCCTCTTCCCTTGAGAAGTCCTACGAACTTCCCGACGGCCA
P. monodon			GAGATCGTGCGCGACGTCAAGGAGAAACTCTGCTACATCGCTCTCGACTTTGAAGGTGAAATGAACGTTGCTGCTGCTTCTTCCTCCCTGGACAAGTCATACGAACTTCCTGACGGCCA
P. uncta			GAGATTGTGCGTGATGTCAAGGAAAAGCTTTGCTATGTCGCCCTCGACTTCGAGAACGAAATGGCTCAGGCTGCTGCTTCCACATCCCTGGACAAGTCATACGAACTTCCCGACGGCCA
S. crassicornis			GAAATTGTTCGTGATGTCAAGGAGAAGCTCTGCTATGTCGCCCTCGACTTCGAGCAGGAGATGACCACCGCTGCTTCCTCCTCCTCCCTCGAGAAGTCCTACGAGCTCCCCGATGGCCA

Table S3. Beta-actin gene sequence of ten different shrimp species using conserved primer. The sequence of PCR amplified fragment by [B-act FM – B-act RE] primer pair.

Shrimp Species with fragment size (bp)	Beta-actin Gene Sequence (Partial)
F. merguiensis (824 bp)	GGATGACATGGAGAAGATTTGGTATCACACTTTCTACAACGAACTCCGTGTCGCTCCCGAAGAGTGTCCTATTCTCCTTACCGAAGCTCCTTTGAATCCTAAGGTTAACCGTGAGAAGATGACGCAGATCATGTTCGAAGTGTTCGCCGCCCCGGCCA<br>TGTACGTGGCCATCCAGGCTGTTCTCTCGCTGTACGCCTCTGGCCGTACCACTGGTATCGTGCTGGACTCGGGCGACGGCGTGTCTCACTGCGTGCCAATCTTTGAAGGGTATGCGCTCCCCCATGCCATCCTCCGACTCGACCTTGCAGGACGCGACTT<br>GACTGACTACCTGATGAAGATCATGACCGAGCGTGGCTACTCCTTCACCACGACTGCCGAGAGGGAGATCGTGCGCGACATCAAGGAGAAGCTCTGCTATGTTGCTCTAGATTTCGAGAACGAGATGGGCGTTGCCGGGGCTTCTTCCTCACTCGACAA<br>GTCTTACGAGCTTCCAGACGGCCAGGTAGTGACCATCGGCAACGAGCGCTTCCGCTGCCCCGAGGCACTCTTCCAGCCTTCCTTCCTCGGTATGGAATGCTCCGGACTCCACGAAACCGTTTACACCTCCATCATGAAGTGCGACGTCGACATCAGGAA<br>GGAGCTGTACGCCAACAACGTCCTGTCTGGAGGCACTACCATGTACCCCGGCATCTCTGACCGCATGCAGAAGGAAATCACTGCCTTGGCTCCCTCCACGATCAAGATCAAGATCATCGCTCCGCCCGAGAGAAAATACTCCGTGTGGATCGGCGGCT<br>CCATCCTCTCCTATCTACCTTCCAGTCCAT
L. vannamei (830 bp)	GGATGACATGGAAAACATTTGGTACCACACCTTTTACAATGAGCTCCGTGTTGCCCCTGAGGAGTCCCCCACACTTCTCACTGAGGCTCCCCTCAACCCCAAGGCCAACCGTGAGAAGATGACTCAGATCATGTTCGAGTCCTTTCCAGGTCCCCGCCA<br>CCTATGTTACCATCCAGGCTGTCCTGTCTCTGTACGCCTCTGGTCGTACCACTGGTCAGGTTTGCGACTCTGGTGATGGTGTAACCCACATGGTCCCCGTCTATGAAGGTTTCGCTCTTCCTCATGCTATCCTTCGTCTCGACTTGGCTGGACGTGACCTGA<br>CCAACTACCTTATGAAGATCATGACTGAGCGTGGCTACTCCTTCACCACCACCGCTGAACGTGAAATCGTCCGTGACATCCAGGAGAAGCTTTGCTACATCGCCCTCGACTTCGAAGGCGAAATGAACGTCGCTGCTGCTTCCTCTTCCATTGATAAGTCTT<br>ATGAACTTCCCGACGGTCAGGTTATCACCATCGGTAACGAGCGTTTCCGTTGCCCTGAGGCTCTGTTCCAGCCTTCCTTCCTTGGTATGGAATCTGCTGGTGTTCAGGAAACCGTCCACAGCTCCATCATGAGGTGCGACATTGACATCAGGAAGGATCTGT<br>TCGCCAACATTGTTATGTCTGGTGGTACCACCATGTACCCTGGTATTGCTGACCGCATGCAGAAGGAAATCACTTGCCTGGCTCCCTCCACCATCAAGATCAAGATTATTGCTCCTCCTGAGCGTAAATACTCCGTCTGGATCGGTGGTTCCATCCTTGCCT<br>CTCCTCTATACTACAACCAATAT
M. ensis (794 bp)	GGAGAATGAGCTCCGTGTTGCCCCCGAGGAGTCCCCCACCCTCCTCACTGAGGCTCCCCTCAACCCCAAGCCCAACCGTGAGAAGATGACTCAGATCATGTTCGAGTCCTTCAGCGTGCCTGCCACCTACGTTTGCATTCAGGCTGTGCTCTCCCTGTACGC<br>CTCTGGTCGTACCACTGGTCAGGTTTGCGACTCCGGTGATGGTGTGACTCACTTCGTCCCCGTCTATGAAGGTTTCGCTCTTCCCCCATGCCATCCTTCGTCTGGATCTTGCTGGTCGTGACCTCACCCACTACCTCATGAAGATCATGACTGAGCGTGGCTAC<br>TCTTTCACCACCACCGCCGAACGTGAAATCGTTCGTGACATCAAGGAGAAGCTCTGCTACATCGCCCTCGACTTTGAATCAGAAATGAGTACCGCTGCTGCCCCCTCTTCTATTGACAAGTCCTACGAACTTCCCGACGGTCAGGTCATCACCATCGGCAACG<br>AGCGCTTCCGCTGCGCTGAGGCCCTCTTCCAGCCTTCCTTCCTTGGTATGGAATCTGCTGGTGTCCAGGAGACTGTCTACAGCTCTATCATGAGGTGCGACATTGACATCAGGAAGGACCTGTTCGCTAACATTGTCATGTCTGGTGGTACCACCATGTACCCC<br>GGTATTGCTGACCGCATGCAGAAGGAAATCACTGCTCTTGCTCCTTCCACCCTGAAGATCAAGATCATTGCTCCTCCCGAGCGTAAGTACTCTGTCTGGATCGGTGGTTCTATCCTGTCTGCTTTCCACCTTCCAACCCAT
M. japonicus (821 bp)	GGACGATATGTTTTACATTTGGTACCACACCTTCTACAACGAACTCCGTGTTGCTCCGGAAGAGTGTCCTATTCTCCTCACCGAAGCTCCTTTGAATCCCAAAGTCAACCGAGAGAAGATGACGCAGATCATGTTTGAGGTTTTCGCCGCTCCGGCCATGTACGT<br>CGCCATCCAGGCTGTTCTCTCGCTTTACGCGTCTGGCCGAACCACGGGCATCGTGCTGGACTCGGGAGATGGTGTGTCTCACTGCGTACCAATTTTTGAAGGCTATGCGCTTCCCCACGCCATCCTCCGACTCGACCTTGCTGGGCGTGACTTGACTGACTACCTGA<br>TGAAGATCATGACCGAGCGTGGCTACTCCTTCACCACGACTGCCGAGAGGGAGATCGTGCGCGACATCAAGGAAAAGCTCTGCTACGTCGCCCTCGATTTCGAGAATGAAATGGGCATCGCCGGTGCTTCGTCCTCCCTCGACAAGTCTTACGAGCTTCCTGACGG<br>CCAGGTGGTGACCATCGGCAACGAGCGCTTCCGCTGCCCAGAGGCACTCTTCCAGCCGTCCTTCTTGGGCATGGAATGCTCCGGACTCCACGAAACCGTCTACACCTCCATCATGAAGTGCGACGTCGACATCAGGAAGGAGCTGTACGCCAACACGTTCTGTCTGG<br>AGGCACCACCATGTACCCTGGTATCGCCGACCGCATGCAGAAGGAGATCACGTCCCTCGCTCCCTCCACCATCAAGATCAAGATCATTGCTCCTCCCGAGCGAAAATACTCCGTGGTGGATCGGCGGCTCCATCCTCTCCTCTCTACTTCAGACAT
M. monoceros (831 bp)	AGACGAGAAGGAGAAGATCTGGCATCACACCTTCTACAACGAACTCCGTGTTGCCCCTGAGAAGTCCCCCACTCTTCTGACTGAGGCTCCCCTCAACCCCAAGGCCAACCGCGAGAAGATGACGCAGATCATGTTTGAAACTTTTGGTATGCCTGCCATGTACGTTG<br>CCATCCAGGCTGTCATGTCGCTCTATGCCTCCGGTCGTACCACTGGCATGGTGTGCGATTCTGGTGATGGTGTAAGTCATCACGTCCCTGTCTTTGAGGGTTATGCTCTTCCTCACGCCATGCTGCGTCTGGACATGGCTGGCCGCGATTTGACCTTGTACCTTATGAAGA<br>TCATGACTGAGCGTGGCTACTCCTTCACCACCACCGCTGAACGTGAAATCGTCCGTGACATCAAGGAGAAGCTTTGCTACATCGCCCTCGACTTTGACAGCGAGATGAATGTTGCTGCTGCCTCCTCTTCCCTGGACAAGTCCTTTGAACTTCCCGACGGCCAGATTAT<br>CACCATCGGTAACGAACGTTTCCGCGCCCCCGAGGCCCTCTTCCAACCTTCCTTCCTGGGCATGGAATGTTCTGGCCTTCACGAGACTGTCTACACAGGCATCATGAGGTGCGACATTGACATCAGGAAGGACCTGTTCGCTAACATTGTCATGTCTGGTGGTTCTACC<br>ATGTACCCTGGTATTGCTGACCGCATGCAGAAGGAAATCACAAACCTTGCACCTCCTTCTCTTCAAAGATCAAGATCATTGCTCCTCCCGAGCGTAAATACTCCGTCTGGATCGGTGGCTCCATCTTGGCTTCCCCTCTCCACCTTCCAATCCAT
M. rosenbergii (824 bp)	GGAGAAGATCTGGCATCACTCCCTCTACAACGAACTTCGAATTGCTCCTGAAGAATCTCCAATCCTGCTGACGGAAGCTCCCCTCAACCCCAAAGGCCAACCGCGAAAAGATGACCCAGATCATGTTTGAAACTTTCTGCACCCCAGCCATGTACGTCGCCATCC<br>AAGCTGTTCTCTCTCTTTATGCCTCTGGCAGAACAACAGGTATCGTACTCGATTCTGGTGATGGTGTCACCCACACTGTCCCCATCTACGAAGGTTATGCCCTTCCTCATGCCATCCTTCGTCTTGACCTAGCTGGGCGTGATTTGACCGCCTACCTCATGAAGATCAT<br>GACTGAGAGAGGCTACTCCTTCACCACCACGGCTGAACGAGAAATCGTTCGTGACATCAAGGAGAAGCTCTGCTACGTCGCCCTCGACTTCGAGAGTGAAATGAACGTATCTGCAGCTTCCTCGTCCTTAGAAAAGTCTTACGAACTCCCCGATGGTCAAGTTATTA<br>CCATCGGTAACGAACGATTCCGTGCCCCAGAAGCTCTCTTCCAGCCTTCCTTCCTGGGTATGGAATCTGTTGGTATCCACGAAACCGTCTACAACTCCATCATGAGGTGCGACATTGATATCAGGGAAGGATCTGTTCGCCAACAATGTGCTCTCTGGTGGTACCACC<br>ATGTACCCTGGTATTGCTGACAGGATGCAGAAGGAAATCACTTCTCTTGCTCCTTCCACCATCAAGATCAAGATCATCGCTCCTCCTGAGAGGAAGTACTCCGTCTGGATCGGCGGTTCCATCTTGGCCTTCTCCTCTGTAACTTGCAATAACAT
P. hardwickii (826 bp)	GGATGACATGGAGAAGATCTGGTACCACACCTTTTACAATGAACTTCGCATTGCCCCCGAGGAGTCCCCAACCCTCCTCACTGAGGCTCCTCTTAACCCAAAAGCCAACCGTGAGAAGATGACTCAGATCATGTTTGAATCTTTCAATGTACCAGCTATGTATGTGTCC<br>ATTCAAGCTGTGTTGTCCTTGTATGCTTCTGGCCGTACGACCGGCTTGGTCTGTGACTCCGGTGACGGCGTTTCTCACATGGTCCCTGTCTACGAGGGCTATGCCCTTCCCCATGCCATTCTCCGCGTCGATCTTGCTGGTCGTGACCTTACCAATTATCTTATGAAGATCA<br>TGACTGAACGTGGCTACTCCTTCACCACCACCGCTGAACGTGAAATCGTCCGTGACATCAAGGAGAAGCTCTGCTACATCGCCCTCGACTTTGAAGGTGAGATGAACGCTGCTGCCGCCTCTTCTTCCCTCGACAAGTCTTACGAACTTCCCGACGGTCAGGTCATCACC<br>ATCGGTAACGAGCGCTTCCGTTGCCCTGAGGCTCTCTTCCAACCCTCTTTCCTGGGTATGGAATCTGTTGGTGTTCACGAAACCGTCCACAGCTCCATCATGAGGTGCGACATTGACATCAGAAAGGACCTGTTCGCCAACATTGTCATGTCTGGTGGCACTACCATGTACC<br>CTGGTATTGCTGACCGCATGCAGAAGGAAATTACCAACTTGGCTCCTTCCACCATCAAGATCAAGATTATTGCTCCTCCCGAGCGTAAGTACTCCGTCTGGATCGGTGGCTCCAATCTGTCTCCTCTGTACCTACCAAACCAT
P. monodon (825 bp)	GGACGACATGGAAAACATCTGGCAACACACCTTCTACAATGAGCTCCGTGTTGCCCCTGAAGAGTCCCCCACACTCCTCACTGAGGCTCCCCTCAACCCCAAGGCCAACCGTGAGAAGATGACTCAGATCATGTTTGAGTCCTTCAGCCTTCCTGCCA<br>TGTACGTGACCATCCAGGCTGTGCTTTCCCTGTACGCCTCTGGTCGTACCACTGGTCAGGTTTGCGACTCTGGTGACGGTGTGACTCACTTTGTCCCCGTCTATGAAGGTTTCGCTCTTCCTCATGCTATCCTTCGTCTCGACTTGGCTGGTCGTGACCTTA<br>CCCACTACCTCATGAAGATCATGACTGAGCGTGGCTATTCCTTCACCACCACCGCTGAACGTGAAATCGTTCGTGACATCAAGGAGAAGCTTTGCTACATTGCCCTTGACTTCGAGAGTGAGATGAATGTTGCTGCTGCTTCCTCCTCCTTGGATAAGTC<br>CTATGAACTTCCCGACGGTCAGGTTATCACCATTGGTAACGAGCGTTTCCGTGCCCCTGAGGCTCTGTTCCAGCCTTCCTTCCTTGGTATGGAATCTGCTGGTGTTCAGGAAACTGTTTACAGCTCCATCATGAGGTGCGACATTGACATCAGGAAGGACC<br>TGTTCGCTAACATCGTCATGTCTGGTGGTACCACCATGTACCCTGGTATTGCTGACCGCATGCAGAAGGAAATCACTGCTCTTGCTCCTTCAACCATCAAGATCAAGATCATTGCTCCTCCTGAGCGTAAGTACTCCGTCTGGATCGGTGGTTCCATCCTGT<br>CTTCTCTCCAACTCCAGGACAT
P. uncta (826 bp)	GGATGACATGGAAAACATTTGGCACCACACCTTCTACAATGAGCTCCGTGTTGCCCCCGAGGAGTCCCCCACCCTCCTCACTGAGGCTCCCCTCAACCCCAAGGCCAACCGTGAGAAGATGACTCAGATCATGTTTGAGTCCTTCAACGTGCCT<br>GCTACCTACATTTGCATCCAGGCCGTGCTCTCCCTGTACGCCTCCGGTCGTACCACTGGTGAGGTTTGCGACTCCGGTGATGGTGTGACTCACTTCGTCCCCGTCTATGAAGGTTTCGCTCTTCCCCATGCCATCCTTCGTCTGGATCTTGCTGGTC<br>GTGACCTTACTCACTACCTCATGAAGATCATGACTGAGCGTGGCTACTCCTTCACCACCACCGCCGAACGTGAAATCGTTCGTGACATCAAGGAGAAGCTCTGCTACATCGCCCTCGACTTCGAGAATGAGATGAACGTTGCTGCTGCCTCCTCC<br>TCCCTCGACAAGTCCTACGAACTTCCCGACGGTCAGGTCATCACCATTGGCAACGAGCGCTTCCGCTGCGCTGAGGCTCTCTTCCAGCCTTCCTTCCTTGGTATGGAGTCTGCTGGTGTCCAGGAGACCGTCCACAGCTCCATCATGAGGTGCGAC<br>ATTGACATCAGGAAGGACCTGTTCGCCAACATTGTCATGTCTGGTGGTACCACCATGTACCCCGGTATTGCTGACCGCATGCAGAAGGAAATCACTGCTCTTGCTCCTTCCACCATTAAGATCAAGATCATTGCTCCTCCCGAGCGTAAGTACTCC<br>GTCTGGATCGGTGGCTCCATCCTGTCTCCTACACAACTTCAAAAATAT
S. crassicornis (818 bp)	GGAGAAGATTTGGCATCACACCTTCTACAATGAACTCCGTGTTGCTCCCGAGAAGAGTGCCAATCTTCTGACTGAAGCCCCAATGAATCCTAAGATTAATCGTGAAAAAACGACACAAATCATGTTCGAGACCTTTGCCGCCCCCGCCATGTAC<br>GTGGCCATCCAGGCCGTGCTGTCCCTGTACGCCTCTGGTCGTACCACTGGTATTGTGCTCGATACAGGTGATGGAGTGACCCATTGTGTACCAATCTTCGAAGGTTATGCTCTTCCTCACGCCATCATGCGTCTCGACTTGGCTGGCCGTGACTTGA<br>CCGCTTACCTGATGAAAATCATGACTGAACGTGGCTACTCCTTCACCACTTCCGCTGAAAGAGAAATCGTCCGTGACATCAAGGAGAAGCTCTGCTATGTCGCCCTCGACTTCGAAAGTGAGATGAATGTCGCCGCGGCCTCTTCTTCACTCGACAA<br>ATCATACGAACTTCCCGACGGTCAAATTGTAACCATAGGTAACGAACGTTTTCGTTGCCCTGAGGCTCTCTTCCAACCTTCCTTCTTGGGGATGGAATGTGCTGGACTCCATGAGACCGTTTACACATCCATCATGAAGTGCGACGTCGACATCAGGA<br>AAGAGCTCTACGCGAACAACGTTCTCTCTGGCGGCACCACCATGTACCCTGGCATCTCCGACCGCATGCAGAAGGAAATCACTGCTCTTGCTCCCTCTACCATCAAGATCAAGATCATTGCTCCTCCTGAGCGTAAATACTCCGTCTGGATCGGTGGTT<br>CCATCCTGTCTGCTCTCGCACCTTCAAGCAAAT

Table S4. The 16SrRNA gene sequence amplified by real-time conserved primers [RT16Sf(N) - RT16Sr]

Shrimp Species	Product Sequence
L. vannamei	TAAGGGGACGATAAGACCCTATAAAGCTTTACAATAAGTTACCTATATTATAAATTGTTAGTATAACTTGAGTTTAGGTAACGTTTGTTGCGTTGGGGCGACGAGAATATAATAAGTAACTGTTCTTAAGTTATTTAATGACAGAAATTTCTGGAAAATTAAT<br>GATCCTCTACTAGAGATCATAAGATTAAGTTACTTTAGGGATAACAGCGTAATC
M. japonicus	TCAGGGGGACGATAAGACCCTATAAAGCTTGACAATAATTTCGTTATATTATAAATTGTTAGTATAACTTGATTTTAACGGGAGTTTGTTTCGTTGGGGCGACGGGAATATAATAAATAACTGTTCTTTTAAATATAATTACAAAAATACTTGGTAAATAATTG<br>ATCCTCTATTAGAGATTAAAAGATTAAGTTACTTTAGGGATAACAGCGTAATC
M. rosenbergii	TAGGGGGACGATAAGACCCTATAAAACTTAATATAATTTAGGCTTAACTTGCGATGTGGGTGAAAAGTAGTTTTGCCTGGTTTATATTTCGTTGGGGAGATGAAGATATAATGAGTAACTGTCTATAAAATTTTATAGCATTGACTAGAATTTGATCCTTCCTTG<br>GGGATTAGGAGAATAAGTTACTTTAGGGATAACAGCGTGATT
S. crassicornis	TGAGGGGACGATAAGACCCTATAAAGCTTTACAATAAGTTAATTAGATTATAAATTGTTAGTATAACTTGGTTTTAGTTATTATTTGTTTCGTTGGGGCGACGAGAATATAATTATATATATAACTGTTCTTTTAAGAAGAAAACAAGGTTAATTGGATTAGAATT<br>GATCCTTTAGTAAAGATTAAAAGATTAAGTTACTTTAGGGATAACAGCGTAAT

Table S5. The Cytb gene sequence amplified by real-time conserved primers [RTCytbF1 – RTCytbR1]

Shrimp Species	Product Sequence
F. merguiensis	ATCCCATTCCACCCCTACTTCACCTTTAAAGACATTACAGGCTTCGTAGTTATACTAGCTGCTCTAACACTTCTAACACTATTAAATCCTTACTTACTAGGGGACCCAGATAACTTCATCCCCGCTAACCCCCTAGTAACTCCCGCTCACATTCAACCAGAATGGTACTTCCTTTT

Table S6. The ND1 gene sequence amplified by real-time conserved primers [RTND1F(N) – RTND1R]

Shrimp Species	Product Sequence
M. japonicus	CAAACTAAATACTGGTGATAAGTAATAAGGAAGAAAATTAGATATAACAGGAAGAGTCTGCTCTTTCGTGAATAACTTAACAGCATCAGCAAAAGGCTGCAACAACCCTATAAAACCCACTTTATTAGGTCCTTTACGAATTTGAATGTAACCAAGAATCT<br>TACGTTCTAATAAAGTTAAAAATGCTAC
P. uncta	CAAACTAAATACAGGAGACAAATAATATGGAAGAAAATTAGATATTACAGGAAGAGTTTGCTCCTTTGTAAAAAGTTTAATTGCATCTGCAAATGGCTGTAACAATCCTATAAACCCCACTTTATTAGGACCCTTACGAATTTGGATATAACCTAAAATCTTT<br>CGTTCTAATAAAGTTAAAAATGCTAC

Table S7. The COX1 gene sequence amplified by real-time conserved primers [RTCOX2F(M) – RTCOX2R(M)]

Shrimp Species	Product Sequence
L. vannamei	CAAATCATAAAGATATTGGAACATTGTACTTTATCTTCGGGGCTTGAGCTGGAATAGTAGGTACCGCTCTTAGATTTATTATCGGAGGGGAACTGTCGTCCCCTGGGGAGCCTCATTGTTGATGATCAA
M. rosenbergii	CAAATCATAAAGACATCGGAACCTTTATACTTTATTTTCGGAGCTTGAGCAGGAATAGTAGGTACAGCTCTTAGTCTTATTATTCGTGCTGAATTAGGTCAACCAGGAAGCCTTTATTGGAGATGATCAA
P. hardwickii	CAAATCATAAAGATATTGGTACCTTATATTTTATCTTTGGAGCTTGATCCGGTATAGTCGGGACTGCTTTAAGTTTAATTATCCGAGCTGAGTTAGGTCAACCAGGTAATCTTATTGGAGATGATCAA
P. monodon	CAAATCATAAAGATATTGGAACTTTATACTTTATTTTCGGAGCTTGAGCAGGAATAGTAGGTACAGCTCTTAGTCTTATTATTCGTGCTGAATTAGGTCAACCAGGAAGCCTTATTGGAGATGATCAA
P. uncta	CAAATCATAAAAGACATCGGAACCCTATATTTTATCTTCGGGGCTTGAGCAGGAATGGTAGGTACGGCTCTCAGACTAATTATCCGGGCCGAACTCGGTCAACCAGGCAATCTTATTGGAGATGACCAA

Table S8. The COX1 gene sequence amplified by real-time conserved primers [RTCOX1F(N) – RTCOX1R]

Shrimp Species	Product Sequence
F. merguiensis	TAACTATAGACCGAATACCTCTTTTCGTCTGAGCGGTATTTATTACAGCCTTACTACTTTTACTATCATTACCAGTTTTAGCGGGAGCTATTACAATGCTTCTAACGGACCGGAACCTAAATACTTCATTCTTCGATCCTGCAGGAGGGGGAGATCCTGTTTTATATCAACACTTATTTTGATTTTTTGGTCACCCTGAAGTTTA
L. vannamei	TAACTATAGGCCGTATACCTCTATTTGTATGAGCAGTATTTATCTCTGCTTTATTATTATTTTTATCATTTCCCGTGTTAGCTGGAGCTGTTTCTATACTTTTAACAGAGCGTAATCTTAACACATCATTTTTCGACCCATCAGGATGAGGAGACCCAGTTTTATATCAACCTTTATTTTGATTTTTTGGTCACCCTGAAGTTTA
M. ensis	TAACAATAGACCGTATACCACTTTTCGTATGAGCTGTATTTATTACAGCCCTACTTCTTCTATTATCTTTACCAGTTTTAGCAGGAGCTATTACCATATTACTAACAGACCGGAATCTTAATACTTCTTTCTTTGACCCAGCAGGAGGAGGTGACCCTATTTTATACCAACATTTATTTTGATTTTTTGGTCACCCTGAAGTTTA
M. japonicus	TAACTATAGACCGAATACCGTTATTCGTCTGAGCAGTTTTTATTACAGCGCTCTTACTCCTTTTATCTTTACCCGTTCTAGCAGGAGCTATTACAATACTTCTTACCGATCGTAATCTTAATACTTCGTTTTTTGACCCAGCAGGTGGGGGAGACCCGGTCCTTTACCAGCATTTATTCTGATTTTTCGGTCATCCTGAAGTATA
M. monoceros	TAACTATGGACCGTATACCACTCTTCGTGTGAGCTGTCTTTATTACAGCCTTGCTATTATTACTATCCCTCCCAGTCCTAGCCGGAGCAATCACTATATTACTAACTGACCGAAACCTTAATACTTCATTCTTTGATCCAGCGGGTGGTGGAGACCCCATCCTCTATCAACATTTATTTTGATTTTTTGGTCACCCTGAAGTTTA
M. rosenbergii	TAGACCGAATACCACTTTTTGTTTGAGCAGTGTTTATTACAGCCCTACTTCTATTACTATCTTTACCAGTCCTAGCAGGAGCTATTACTATACTGTTAACAGATCGTAATTTAAATACATCATTCTTTGACCCAGCAGGGGGTGGTGACCCTGTTTTATATCAACATTTATTTTGATTTTTCGGTCATCCTGAAGTATA
P. hardwickii	TAACTACAGTAATCAACATACGATCATCTGGGATGACAATAGATCGTATACCTTTATTCGTCTGATCAGTTTTTATCACCGCCTTATTACTACTCCTCTCCCTTCCAGTTCTAGCTGGAGCTATTACTATACTTCTTACTGACCGAAATCTAAATACATCTTTCTTTGACCCTGCCGGAGGTGGTGATCCAATCCTCTACCAACATTTATTTTGATTTTTTGGTCACCCTGAAGTTTA
P. monodon	TAACTATAGACCGAATACCACTTTTTGTTTGAGCAGTGTTTATTACAGCCCTACTTCTATTACTATCTTTACCAGTCCTAGCAGGAGCTATTACTATACTGTTAACAGATCGTAATTTAAATACATCATTCTTTGACCCAGCAGGGGGTGGTGACCCTGTTTTATATCAACATTTATTTTGATTTTTTGGTCACCCTGAAGTTTA
P. uncta	TAACCACAGTAATCAACATACGATCTTCAGGAATATCGATGGACCGCATACCCTTGTTTGTATGGTCAGTATTCATTACTGCTCTTCTCCTTCTTCTCTCTTTACCAGTCTTAGCAGGGGCTATCACAATACTCTTAACAGACCGTAACTTGAATACATCTTTCTTTGACCCGGCCGGAGGAGGGGACCCTATCCTCTATCAACACTTGTTTTGATTTTTCGGTCATCCTGAAGTATA
S. crassicornis	TATTAATAGAACGAATATCCCTTTTTGTTTGATCCGTATTTATCACAGCTATTCTCTTATTACTTTCATTGCCCGTATTAGCTGGAGCAATTACTATACTTTTAACTGATCGAAACTTTAATACTACATTCTTTGACCCAAGAGGAGGGGGAGATCCTATTTTATATCAACACTTATTTTGATTTTTTGGTCACCCTGAAGTTTA
